# Distribution, Site-Specific Water Quality Criteria, and Ecological Risk Assessment of Heavy Metals in Surface Water in Fen River, China

**DOI:** 10.3390/toxics11080704

**Published:** 2023-08-15

**Authors:** Huixian Li, Yue Li, Guanghui Guo, Yang Li, Ruiqing Zhang, Chenglian Feng, Yahui Zhang

**Affiliations:** 1 State Key Laboratory of Environmental Criteria and Risk Assessment, Chinese Research Academy of Environmental Sciences, Beijing 100012, China; lihuix111@126.com (H.L.); fengchenglian@163.com (C.F.); 2 School of Ecology and Environment, Inner Mongolia University, Huhhot 010021, China; iyuuue1999@163.com; 3 Institute of Geographic Sciences and Natural Resources Research, Chinese Academy of Sciences, Beijing 100101, China; kellyggh@163.com; 4 College of Water Resource Science and Engineering, Taiyuan University of Technology, Taiyuan 030030, China; happyyang211@163.com; 5 Environmental Analysis and Testing Laboratory, Chinese Research Academy of Environmental Sciences, Beijing 100012, China

**Keywords:** heavy metals, ecotoxicity, water quality criteria, ecological risk assessment, Fen River

## Abstract

Due to a lack of toxicity reference values that match the regional environmental characteristics, the ecological risk of metals in water bodies cannot be accurately assessed. The Fen River is the second-largest tributary of the Yellow River in China, and the sustainability of this area is threatened by heavy metal pollution caused by intensive industrial and agricultural activities. In this study, site-specific water quality criteria (WQCs) for heavy metals in the Fen River were derived considering toxicity data from native aquatic organisms and regional water quality factors (e.g., water hardness). Short-term WQCs for Mn, Cu, Cd, Zn, Cr, Pb, and Ni were 2026.15, 98.62, 10.02, 63.07, 6.06, 166.74, and 132.73 μg/L, respectively, and long-term WQCs were 166.53, 29.71, 2.18, 19.29, 4.15, 6.38, and 14.76 μg/L, respectively. The distribution characteristics of these metals during the wet season in 2020 were explored, and their average concentrations in the river water did not exceed the environmental quality standards for surface water in China but were higher than the world average levels. Cr was the main pollutant in the sampling sites of Yaodu region, Hongdong Shitan, Xiao River, and Duanchun River, as was Pb in Duanchun River. Based on the site-specific WQCs, using hazardous quotient (HQ) and margin of safety (MOS_10_) approaches, a high risk of Pb was identified in the Duanchun River, and a medium risk of Cr might occur at midstream and downstream of Yaodu and Xiaodian. The results will provide a reference basis for heavy metal pollution control and water quality management in the Fen River.

## 1. Introduction

Heavy metal pollution has attracted global attention because of its significant biological toxicity, persistence, and typical accumulation over many years [1,2]. With the rapid development of industry and agriculture in China over the past three decades, large amounts of pollutants, including heavy metals, have been released into the natural water environment [3]. The accumulation of heavy metals in aquatic environments poses serious risks to aquatic ecosystems [4] and human health [5,6]. In recent years, heavy metal pollution incidents have occurred frequently, and they pose potential threats to the ecological security of river basins in China [7]. The European Union Water Framework Directive [8] requires a good chemical and ecological status of European surface waters by the end of 2015. The European Commission’s objective of the attainment of good chemical status aims to deal with chemical pollution of surface waters, as chemical pollution can have an impact on aquatic ecosystems (e.g., by causing loss of biodiversity) and may hinder the production of clean drinking water. To define good chemical status, environmental quality standards have been established for chemical pollutants of high concern, especially heavy metals, and chemical effects monitoring in surface waters is currently conducted. Monitoring the chemical status of water bodies is crucial to assist environmental policy, identify chemical fingerprints, and further reduce source-orientated pollutants. At present, it is still necessary to pay enough attention to heavy metal pollution and its toxic risks.

Shanxi Province has a coal reserve accounting for 1/4 of the total coal reserves in China, and the resultant massive exploitation of coal mines has led to environmental problems such as soil erosion. Further, increasing industrial sewage and agricultural irrigation have been deteriorating the water quality [9]. The Fen River (FR) is facing significant challenges from anthropogenic pollution from industrial and municipal domestic sources and changes in river water quality, especially heavy metal pollution [9,10,11]. For example, the concentration of Hg, Cr, and Cd at downstream Hejin exceeded the Grade Ⅴ standard of environmental quality standards for surface water (GB3838-2002) in 2005 [12]. Zhang [13] found that Cd, Cr, Pb, and Cu pollution were relatively serious at each sampling point in the upper reaches of FR, and the content of these metals at individual sites exceeded the Grade V standard values in 2011. Xiao et al. [14] conducted a preliminary study on the water quality of FR based on the trace elements and found that As, Cr, and B were the main pollutants. Due to anthropogenic input, the water quality in the middle stream was found to be the worst, and As, Mn, Ni, Ba, Se, and V were potential pollutants that posed a health risk [11]. 

Based on a recent study, it appears that the water quality in the Fen River Basin has been improving in recent years [15]. However, all these previous studies focused on the pollution level of individual heavy metals and water quality evaluation in different sections of the Fen River Basin [12,16,17,18,19]. The current status of pollution characteristics and ecological risk assessment of heavy metals along the whole River is still not clear, and this information is urgently needed for future water risk management. Moreover, it is difficult to accurately evaluate the potential hazard risk of heavy metals to aquatic organisms without regional water quality criteria (WQC) for the FR basin. Site-specific WQC is the main reference basis for regional water quality management and ecological risk assessment. Water quality parameters (e.g., hardness, Dissolved organic carbon (DOC), alkalinity, pH, etc.) can affect the toxicity of metals to aquatic organisms [20,21,22]. Due to water quality parameters and species composition, WQC may be different at the national and regional levels [23,24]. Site-specific WQCs are based on the characteristics of regional water chemistry and toxicity data from native species, and they can provide better protection for aquatic organisms from the toxic effects of pollutants [25,26]. The geographical and climatic characteristics of the FR basin are unique, and high values of pH, alkalinity, and hardness were detected in the water [27], but there is no site-specific WQC available for the risk assessment in this basin area. To understand the toxicity risk of high-level heavy metal pollution in FR, it is necessary to establish site-specific WQCs of typical metals according to the water quality parameters and taxonomic groups and conduct qualitative and quantitative ecological risk assessments (ERA). At present, the ERA of heavy metals in the FR Basin is mostly based on the risk analysis of pollution levels and geological indicators, and the possible toxic effects of metals on aquatic organisms were not considered. The hazardous quotient (HQ) method is the most commonly used for a preliminary assessment of ecological risk because of its simplicity [28]. Probabilistic assessment, such as the margin of safety (MOS_10_), was used to quantify the likelihood of toxic effects occurring, in which both the toxicity distribution curve and exposure concentration curve of chemicals were used [29]. 

The aims of this study were: (1) to detect and analyze the content and distribution characteristics of seven typical metal elements (Cr(Ⅵ), Mn, Ni, Cu, Zn, Cd, and Pb) in the water of FR and to evaluate the pollution level of these metals by using the pollution index method; (2) to derive site-specific WQCs of these metals to protect aquatic life in FR; and (3) to qualitatively and quantitatively assess the ecological risk assessment of heavy metals to aquatic organisms. The results of this study could provide a reference for regional heavy metal pollution control and water quality management.

## 2. Material and Methods 

### 2.1. Overview of the Survey Region

**Figure 1:**
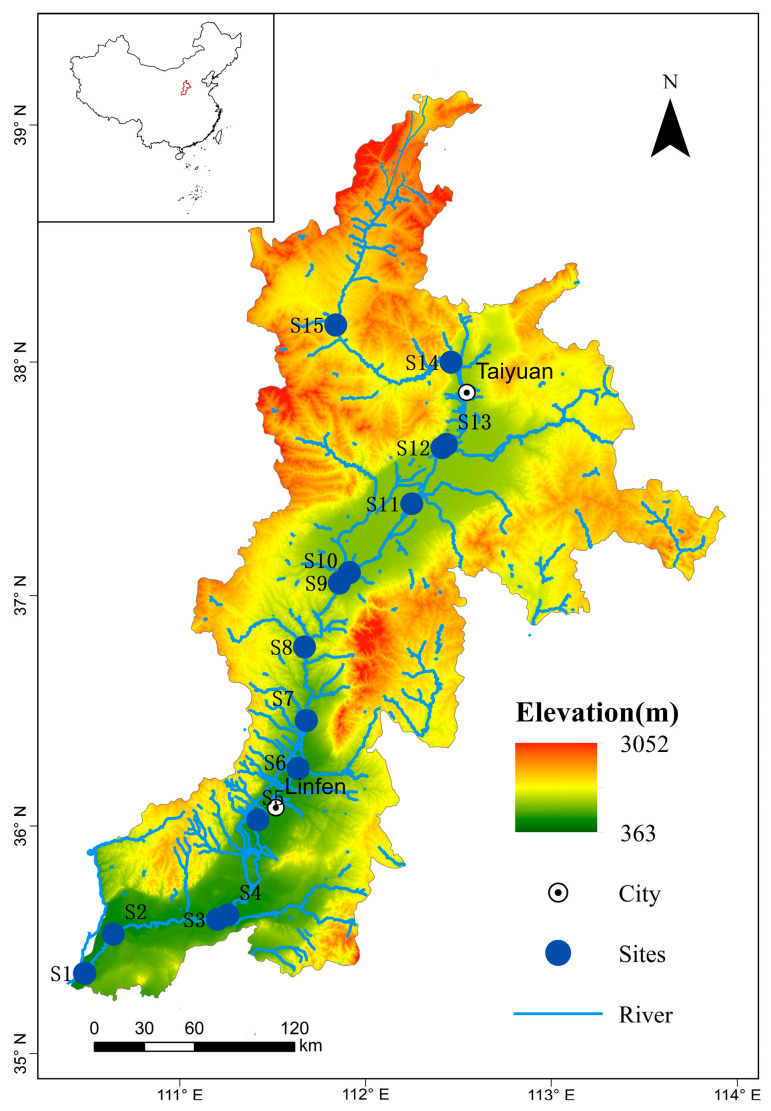
Sampling sites in the Fen River in Shanxi Province.

The FR (35°–39° N, 110°–113° E) is 713 km long and covers an area of 39,721 km^2^ in the eastern Loess Plateau in China [30] (Figure 1). The FR is the second-largest tributary of the Yellow River in China and is the largest ecological corridor in Shanxi Province. Its water environment quality not only has an important impact on the water ecology of the Yellow River basin but also relates to the overall ecological pattern and socio-economic development of Shanxi Province. The upstream section of the FR is from the source to the Lancun hydrological station, with a channel length of 217 km. The midstream is from the Lancun hydrological station to the Shitan hydrological station, with a channel length of 285 km. The section after Shitan hydrological station is downstream, with a channel length of 211 km. The Fen River basin has a mid-latitude continental monsoon climate with an annual average precipitation of 465 mm, which is mainly concentrated from July to September [15]. Coal, bauxite, refractory clay, iron ore, pyrite, and oil shale are the main mineral resources of the Fen River basin. Coal mines are mainly distributed in the county of Ningwu and the cities of Taiyuan, Jincheng, and Linfen, and pyrite ore is mainly distributed in the cities of Changzhi and Jincheng, as well as the county of Hejin [11]. As a seasonal river, the FR is one of the most polluted rivers in the Yellow River basin of China [31]. In 2017, the discharge of waste water reached 337 million tons in the FR basin. Among the 117 sewage outlets, industrial and mining enterprises account for about 1/3 of the total, leading to increasingly serious water pollution problems in the Fen River basin [32]. The area of the Fen River Basin accounts for 25% of the total area of the province, involving 9 cities and 51 counties (cities and districts). By the end of 2018, the permanent resident population of administrative regions in the Fen River Basin was 144.578 million. Extensive cropland is distributed on both sides of the FR. The agricultural plantings are mainly distributed in the mountains, hills, and basin plains. The middle and lower reaches are also the main agricultural production areas. The FR, as the largest river in Shanxi Province, receives more than 100 tributaries from its source to the estuary of the Yellow River. 

### 2.2. Collection and Analysis of Water Sample

Water samples were collected in September 2020 at fifteen sites (Figure 1). The sites were Wanrong (S1), Hejin (S2), Hui River (S3), Houma (S4), Yaodu District (S5), Honganjian River (S6), Shitan (S7), Duanchun River (S8), Wenyu River (S9), Ciyao River (S10), Changyuan River (S11), Xiao River (S12), Taiyuan (S13), Yangxing River (S14), and Lan River (S15). The S1~S7 belong to the downstream, and the S8~S14 are midstream. S15 is upstream of the river. 

All the river water samples were filtered through a 0.45 μm Whatman^®^ nylon filter(Whatman, Manchester, UK) at the sampling site. The filtered 60 mL water samples were stored in cleaned and acidified Nalgene bottles for trace element analysis, and the 30 mL samples were stored in cleaned and unacidified Nalgene bottles for anion analysis. The temperature and pH were determined by a water quality parameter instrument (HACH Hydrolab DS5, Loveland, CO, USA). The concentrations of trace elements and major cations (Ca^2+^, Mg^2+^, Na^+^, and K^+^) in river water were detected by ICP-MS (Agilent 7500a, Santa Clara, CA, USA). The anions (SO_4_^2−^ and Cl^−^) were analyzed by ion chromatography (Thermo ICS-2100, Waltham, MA, USA). The DOC in water samples was analyzed by a total organic carbon analyzer (O.I. Aurora 1030c, Danvers, MA, USA). The hardness of water samples was determined by the hardness digital titration (HACH 16900, Loveland, CO, USA), and the alkalinity was determined by using the portable test instrument (HACH HQ40d, Loveland, CO, USA). Blank samples were spiked and provided in parallel for quality control. If the error between repeated reference material analysis and repeated sample analysis was less than 5%, the result was considered reliable. The precision of the repeated sample analysis was less than 10%.

### 2.3. Water Quality Assessment

The single-factor pollution index method was used as an indicator for water quality assessment (Equation (1)) [33].

(1) $$P_{i}=p_{i}/S_{i}$$

where *P_i_* is the single factor pollution index of element *i*, referring to the pollution level of single heavy metals; *p_i_* is the mass concentration of element *i*; and *S_i_* is the standard value of element *i*, referring to the environmental quality standard for surface water (GB 3838-2002) [34,35]. The evaluation criteria for the single factor pollution index are shown in Table 1. 

The integrated pollution index (*I*) was used to evaluate the comprehensive pollution level of seven heavy metals (Equation (2)). The evaluation standard for the integrated pollution index is shown in Table 1 [33].

(2) $$I=\frac{1}{n}\sum_{i=1}^{n}P_{i}$$

where *I* is the comprehensive pollution index, *n* is the number of elements, and *P_i_* is the single factor pollution index of element *i*.

### 2.4. Derivation of Water Quality Criteria

#### 2.4.1. Toxicity Data Collection and Screening

The acute and chronic toxicities of Cr, Mn, Ni, Cu, Zn, Cd, and Pb on native species found in FR were collected from commonly used databases, including ECOTOX (http://cfpub.epa.gov/ecotox (accessed on 11 August 2022)), the China Knowledge Resource Integrated Database (http://www.cnki.net (accessed on 20 September 2022)), and published literature and reports. At the same time, water chemical parameters related to toxicity values were also sorted out for deriving site-specific WQCs. Water hardness was collected for toxicity data for Mn, Ni, Zn, Cd, and Pb, and pH values were for Mn. For Cu, the toxicity data with major cations, anions, and DOC were collected according to the US EPA copper document [20]. 

The data screening method was referenced in the technical guideline for deriving water quality criteria for freshwater organisms (HJ 831-2022) [36] with the following basic principles: (1) The tested species exist in China, excluding harmful invasive species. (2) Single-celled animals and microorganisms (except microalgae) were discarded. (3) The purity of the test material was ≥95%. (4) The acute toxicity endpoints were mainly the median effect concentration (EC_50_) and the median lethal concentration (LC_50_), with an exposure time of ≤4 days. The chronic toxicity endpoints were the maximum acceptable toxicant concentration (MATC), 20% effective concentration (EC_20_), 10% effective concentration (EC_10_), no observed effect concentration (NOEC), and lowest observed effect concentration (LOEC). Preference was given to the chronic toxicity data with exposure durations of 21 days or across at least one generation. (5) Toxicity data for sensitive life stages were preferred. (6) Toxicity data with measured concentrations of the test material were preferred to nominal concentrations. The geometric mean was used if there were multiple toxicity data for one species. 

#### 2.4.2. Toxicity Data Processing and Derivation of Water Quality Criteria

The influence of factors was accounted for by using a covariance analysis with toxicity values and hardness or pH [37,38]. The selected data, including hardness values, needed to meet the criteria that definitive toxicity values were available over a range of hardness concentrations, where the highest hardness was higher than 3 times the lowest and over 100 mg/L higher than the lowest value. The toxic concentrations of manganese in algae were standardized for different pH values [22]. For each selected toxicity value, including pH, the amount needed to meet the pH spanned at least 1.5 units. For Cu, the BLM method was recommended by the US EPA for the derivation of copper WQC [20]. There were insufficient data to develop an algorithm based on water factors for Cr(VI), so the WQCs of Cr were directly derived by using the Species Sensitivity Distribution (SSD) curve method without standardization.

The acute toxicity pooled hardness slope was calculated using Equation (3) and the acute toxicity after hardness normalization was calculated using Equation (4). The chronic toxicity pooled hardness slope was calculated using Equation (5) and the chronic toxicity after hardness normalization was calculated using Equation (6). The chronic toxicity pooled pH slope of Mn was calculated by Equation (7), and the chronic toxicity after pH normalization was calculated using Equation (8).

*ln*(*ATV*) = *K_A_ln*(*H_A_*) + *C_A_* (3)

(4) $${ATV}_{H}=e^{\left((\left(\ln\left(ATV\right)\right)+K_{A}\times \left(H_{A}-H\right)\right)}$$

*ln*(*CTV*) = *K_C_ln*(*H_C_*) + *C_C_* (5)

(6) $${CTV}_{H}=e^{\left((\left(\ln\left(CTV\right)\right)+K_{C}\times \left(H_{C}-H\right))\right)}$$

*ln*(*CTV*) = *K_pH_ln*(*pH_C_*) + *C_C_* (7)

(8) $${CTV}_{pH}=e^{\left(\left((\ln\left(CTV\right)\right)+K_{pH}\times \left({pH}_{C}-pH\right))\right)}$$

where *ATV* is the acute toxicity value before modification, μg/L; *CTV* is the chronic toxicity value before modification, μg/L; *ATV_H_* is the acute toxicity value after hardness modification, μg/L; *CTV_H_* is the chronic toxicity value after hardness modification, μg/L; *CTV_pH_* is the chronic toxicity value after pH modification, μg/L; *k_A_* is the slope for the *ln–ln* relationship between acute toxicity and water hardness; *k_C_* is the slope for the *ln–ln* relationship between chronic toxicity and water hardness; *k_pH_* is the slope for the *ln–pH* relationship between chronic toxicity and pH values; *H_A_* is the water hardness before acute toxicity modification in mg/L; *H_C_* is the water hardness before chronic toxicity modification in mg/L; *pH_C_* is the pH before chronic toxicity modification; *C_A_* is the acute toxicity value intercept constant; *C_C_* is the chronic toxicity value intercept constant; and H is the water hardness (g CaCO_3_/L).

The normal distribution for modified toxicity values was tested using the K-S test. The averages of normalized toxicity values were then sorted from low to high, and the species accumulative probabilities were calculated using Equation (9).

(9) $$P=\frac{R}{N+1}$$

where *P* is the cumulative probability; *R* is a sequence number; and *N* is the maximum number.

Toxicity values and accumulative probabilities were used to construct the SSD curve based on four models, including logistic, log-logistic, normal, and log-normal models. A software of China-WQC 2.0 (https://www.mee.gov.cn/ywgz/fgbz/hjjzgl/mxrj/202203/t20220304970658.shtml (accessed on 11 November 2022) was used for SSD analysis. The hazardous concentrations of 5% of the species affected (HC_5_) were calculated from the SSD curves. The WQCs were calculated as HC_5_ divided by an assessment factor of 2 [36]. The LWQC can be calculated by dividing the HC_5_ by the acute-to-chronic ratio (ACR) if chronic toxicity data are lacking [37]. 

### 2.5. Ecological Risk Assessment

The potential ecological risk of metals was assessed using a tiered approach. The HQs were calculated using the environmental exposure concentration (EEC) of these metals in water divided by WQCs [39]:

HQ = EEC/WQC(10)

When HQ < 0.1, there is no obvious ecological risk; when 0.1 ≤ HQ < 1, there is low risk; when 1 ≤ HQ < 10, there is moderate risk; and when HQ ≥ 10, there is high risk [40,41]. 

In the safety threshold method, all the exposure and toxicity data were used to qualitatively estimate the hazard risk of metals, and MOS_10_ was calculated as the ratio of SSD_10_ to ECD_90_ (Equation (11)). A log-logistic function was used to fit the toxicity data and the environmental exposure data. The modified toxicity data was used in the construction of the SSD curve. If there was no available chronic toxicity data, the acute toxicity was transformed by the acute/chronic data ratio into chronic toxicity (ACR = 100) [42,43].

MOS_10_ = SSD_10_/ECD_90_ (11)

where SSD_10_ is the critical value at 10% of the cumulative probability distribution of the toxicity SSD curve, and ECD_90_ is the critical value at 90% of the cumulative probability distribution of environmental exposure concentrations of the heavy metals. MOS_10_ values less than 1 indicate a high risk to the aquatic organism. Meanwhile, MOS_10_ values higher than 1 indicate low risk to aquatic organisms [40]. 

## 3. Results and Discussion 

### 3.1. Distribution Characteristics of Heavy Metals Pollution

The evaluation of heavy metal pollution levels and water quality in FR is shown in Table 2. The average concentrations of each element decreased in the following order: Pb > Mn > Ni > Cr > Zn > Cu > Cd. Comparing to the results in previous studies (Table 3; [11,12,15]), except for Pb and Ni, the concentrations of Mn, Cr, Zn, Cu, and Cd showed a decreasing trend from 2005 to 2020. It appeared that the water quality in the Fen River Basin has improved in recent years. This might be attributed to a series of environmental protection policies in Shanxi province. The government of Shanxi Province issued the Ecological Restoration Plan in the Fen River Basin (2015–2030), Regulations of Shanxi Province on Ecological Restoration and Protection in the Fen River Basin (2018), and Overall Plan for Ecological Protection and Restoration of “seven rivers” in the Fen River Basin (2018). However, the concentrations of these metals in FR were higher than the world average levels (Table 3). The concentration of Ni in the FR was slightly higher than that in other rivers in China. The contents of Cu, Zn, and Mn were less than those in other rivers, but the levels of Pb and Cr were greater than those in most water bodies. Cd was at a low level, close to the world average level.

**Figure 2:**
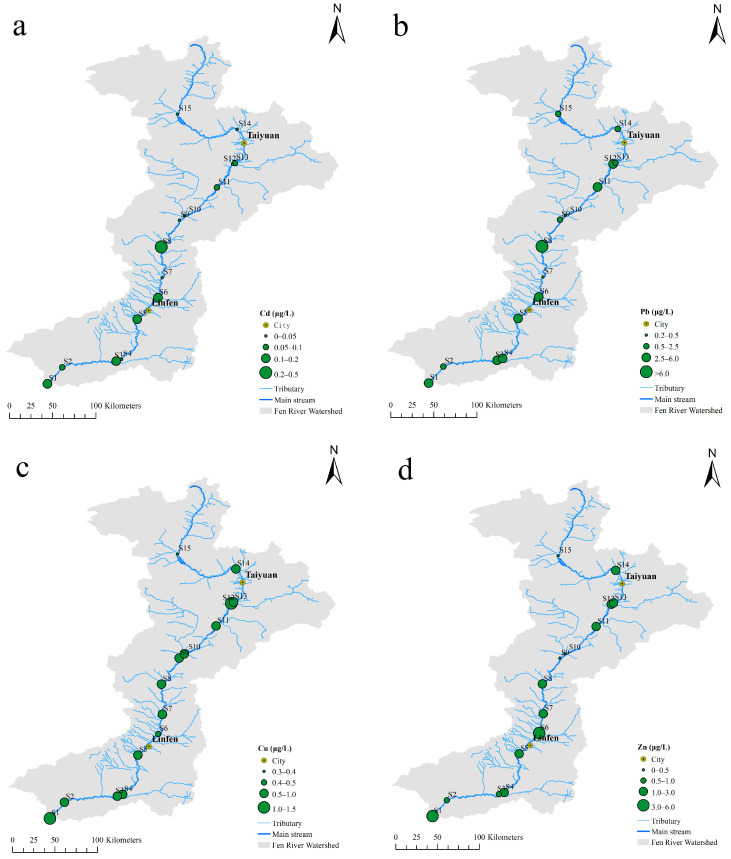

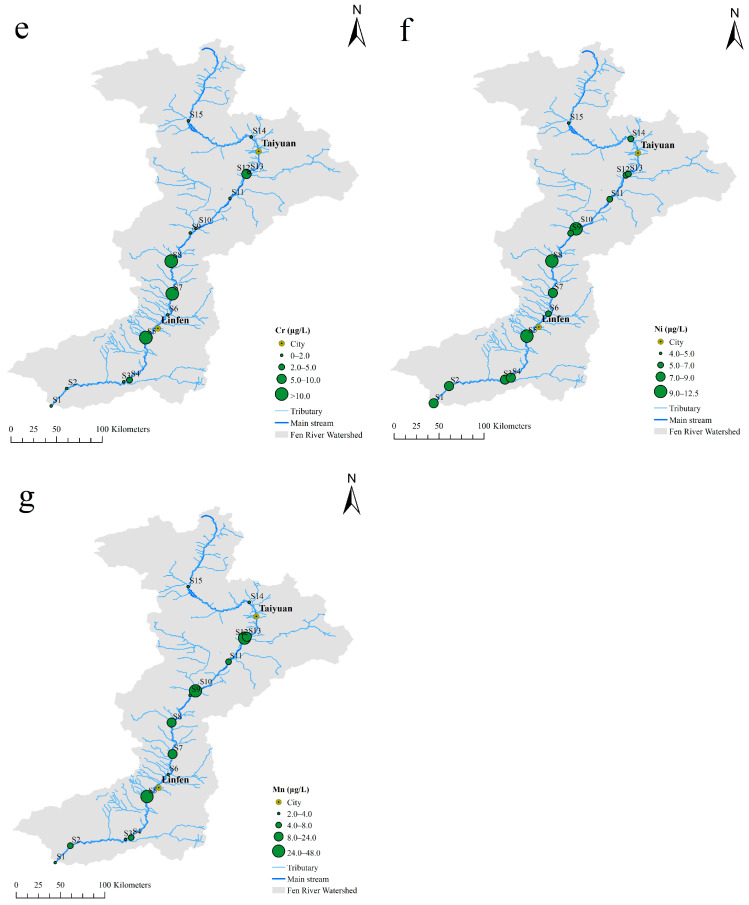
Distribution of heavy metals in the water from the Fen River (**a**–**g** are the heavy metal elements Cd, Pb, Cu, Zn, Cr, Ni and Mn, respectively).

The spatial variation of heavy metals is obvious, with low values upstream, intermediate values downstream, and very high values midstream (Figure 2). The heavy metal content at S15 sampling sites in the upstream is lower than that at other sites, which may be because the upstream of the Fen River is a water source protection area. The very low concentrations in µg/L at the S15 site were 0.02, 1.85, 0.67, 1.96, 1.31, 5.80, and 2.95 for cadmium (Cd), lead (Pb), copper (Cu), zinc (Zn), chromium (Cr), nickel (Ni), and manganese (Mn), respectively. Conversely, heavy metal contamination sections midstream, due to an anthropogenic contribution, are highlighted in many areas characterized by urban settlements and industrial areas. The enrichment factor of these elements is 2–137 times higher than the clean sections upstream. As the geographical location gradually moves southward, it may be due to the inflow of tributaries along the main stream of the Fen River, which leads to an increase in the amount of pollution received by the Fen River and a gradual deterioration of water quality [50].

The concentrations of Pb and Mn in the water samples from the midstream region were higher than those of the downstream and upstream regions (Table 2), which was consistent with the results in the Chai et al. [11] study. The contents of Cd, Cu, Zn, Cr, and Ni had similar distribution characteristics in various regions, and the concentrations increased successively from the upstream, midstream, and downstream. The concentration variation coefficients of Cd, Pb, Cr, and Mn exceeded 100%. The maximum concentrations of Cd and Pb were in the Lingshi region (S8) (Figure 2), and they were 0.50 and 251.18 µg/L, respectively. The spatial distribution of Cu and Zn was relatively uniform. The maximum values of Cu and Zn appeared at the site of Wanrong region (S1) in the lower reaches of FR, and the values were 1.36 and 5.72 µg/L, respectively. The high Cr content area was mainly concentrated in Yaodu section (S5), and the maximum value was 17.12 µg/L. The maximum values of Ni and Mn appeared in the Jiexiu section (S10), and the maximum values were 12.18 and 47.72 µg/L, respectively. The concentrations of Cr in Duanchun River (S8) of Lingshi section in the middle reaches, Yaodu District (S5), and Shitan (S7) in the lower reaches exceeded the Grade II standard limit (50 µg/L) for protection of the habitats of rare aquatic organisms, spawning grounds of fish and shrimp, and feeding grounds for juvenile fish. The lead level in the Lingshi sampling site (S8, 251.18 μg/L) was more than 2.5 times over the Grade III standard limit for protecting the winter migration channel of fish and shrimp and the aquaculture area. and exceeded the standard limit of Grade Ⅴ (100 μg/L) for protection of agricultural irrigation and landscape. The Lingshi sampling site (S8) was located in Lingshi County, and it is close to the industrial area. The leading industries include coal, coal washing, electric power, metallurgy, etc., and industrial emissions may be the main sources of Pb pollution.

**Figure 3:**
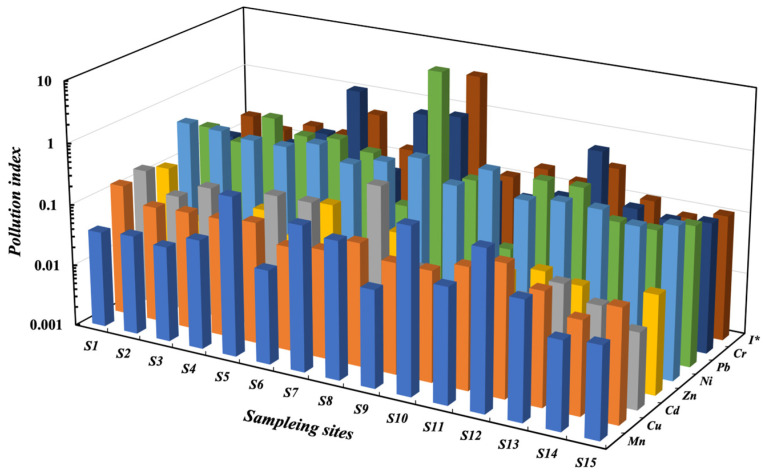
Pollution index of heavy metals in the Fen River (I*—integrated pollution index).

The *P_i_* values of Cd, Cu, Zn, Ni, and Mn at all sampling sites were less than 1, which indicated clean levels (Figure 3, Table S1). However, the *P_i_* values of Cr at the sampling sites of Yaodu (S5), ShiTan (S7), and Duanchun River (S8) were greater than 1, which indicated light pollution in these sites. The *P_i_* values of Pb at S8 in the Lingshi region reached 25.36, which was heavy pollution. The *I* values of Pb at the site (S8) were greater than 4, indicating the level of heavy pollution, and the *I* values at other sites were less than 1. The order of *I* values was: S8 > S5 > S12 > S7 > S3 > S10 > S1 > S4 > S6 > S11 > S2 > S13 > S15 > S9 > S14. The *I* values in the middle and lower reaches (4.9341 ± 1.4577, 1.5652 ± 0.1178) were generally higher than those in the upper reaches (0.1082), especially the middle reaches, which had the highest *I* value. It had been shown in previous studies that the water quality in the middle reaches of FR was the worst due to the industrial waste input, just where the highest concentration of industries occurs [11,15]. Pb and Cr were the main contributing factors to the water pollution of the Duanchun River, and Cr for the Yaodu District in the lower reaches.

Wang et al. [50] conducted an investigation and study on the community structure of benthic organisms in the upper and middle reaches of the Fen River basin, their relationship with water quality and environmental factors, and the diversity of large benthic species. The results showed that the sampling points located at the source of the Fen River had good water quality and other environmental factors and had the highest number of benthic animal species. With the changes in the living environment of large benthic animals, especially the increase in chemical index pollution, their species decreased. The results indicate that the species of macrobenthic animals in the upper reaches of the Fen River are superior to those in the middle reaches. The results of this study on spatial monitoring of heavy metal concentrations in the Fen River also exhibit the same pattern. However, a lack of clear cause–effect relationships between environmental concentrations of heavy metals and ecotoxicological effects or ecological status at many sites under investigation has not been demonstrated in this study. Therefore, other environmental factors, such as heavy metal content, need to be considered as factors affecting the biological status in future research. 

### 3.2. Hardness and pH Dependent WQCs

**Figure 4:**
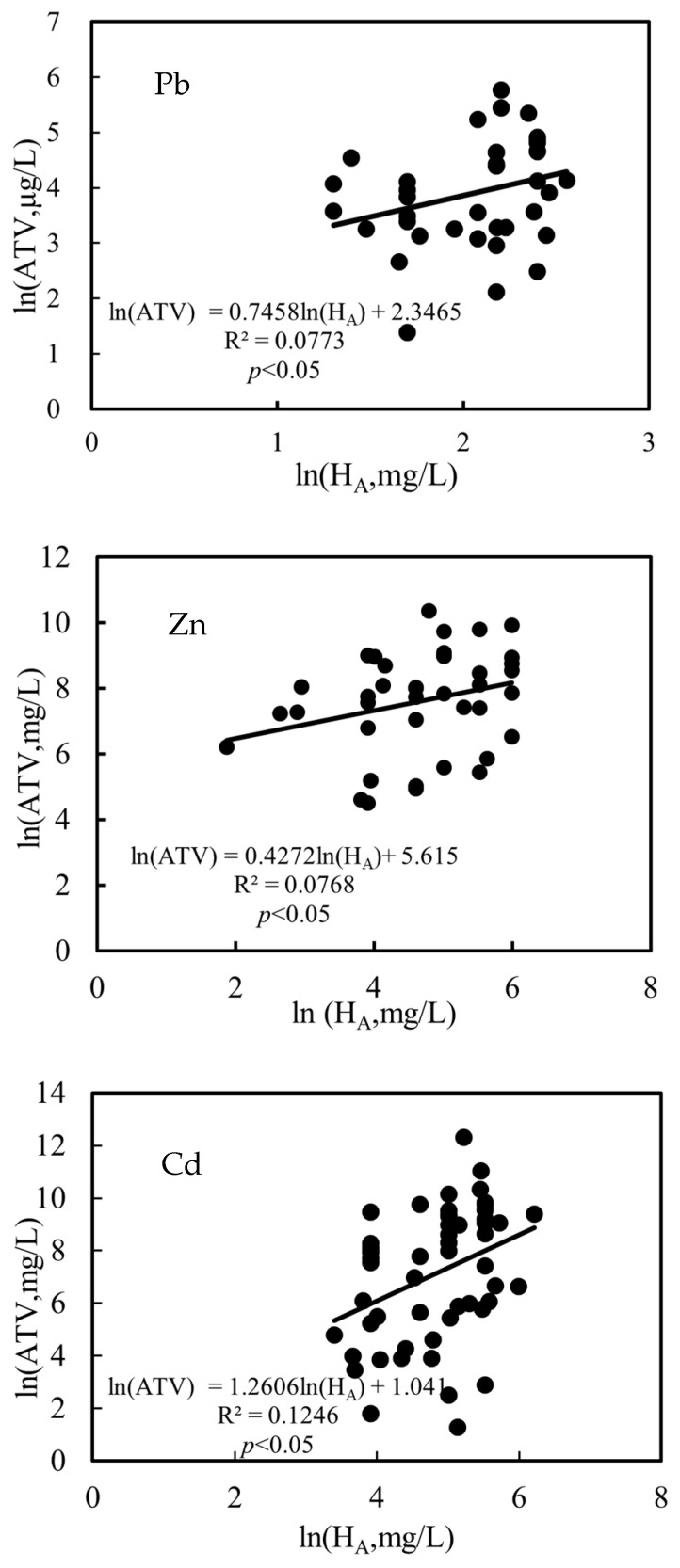

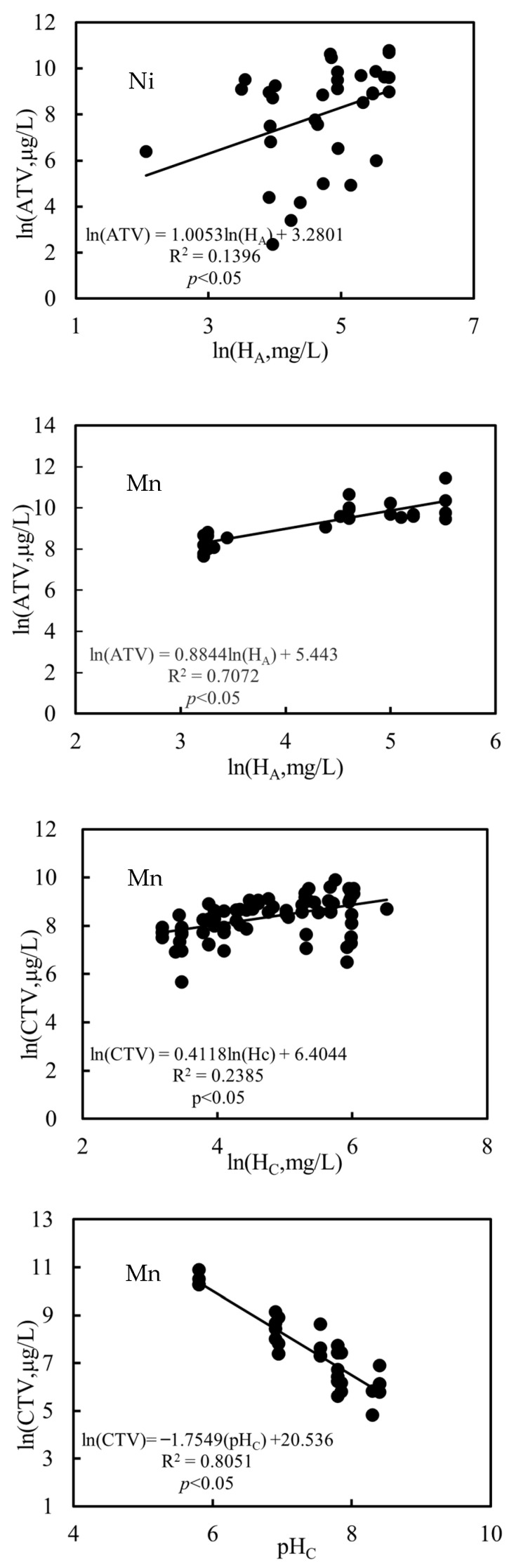
The linear regression fit between water hardness, pH and toxicity to aquatic organisms.

The acute and chronic toxicity data collected are shown in Tables S2–S9 in the Supplementary Materials. The pooled slopes for the ln–ln or ln–pH relationship and the fitting equation between organism toxicity and the water parameter of these metals are shown in Figure 4.

The water quality parameters of samples from 15 sampling sites in the FR are shown in Table S10. The median values of the water quality parameters in the FR were as follows: temperature = 21.1 °C, pH = 8.31, hardness = 182.66 mg/L (CaCO_3_), DOC = 8.82 mg/L, Ca^2+^ = 59.77 mg/L, Mg^2+^ = 24.65 mg/L, Na^+^ = 40.34 mg/L, K^+^ = 7.39 mg/L, SO_4_^2−^ = 230.30 mg/L, Cl^−^ = 93.47 mg/L, alkalinity = 267.12 mg/L. HA and S^2−^ were set to their default values, which were 10% and 1 × 10^−10^ mg/L, respectively. The pooled slopes of these metals were used to normalize the toxicity data to a hardness of 180 mg/L CaCO_3_ or pH 8.30, which were the approximate water parameters of FR. The SWQCs of Mn, Cu, Cd, Zn, Cr, Pb, and Ni in FR derived by SSD method were 2026.15, 98.62, 10.02, 63.07, 6.06, 166.74, and 132.73 μg/L, respectively (Table 4). Since there were few effective chronic data of Cu, Cd, Zn, Cr, Pb, and Ni obtained in this study, the acute chronic ratios (ACR) were used to derive LWQCs. The LWQCs of Mn, Cu, Cd, Zn, Cr, Pb, and Ni were 166.53, 29.71, 2.18, 19.29, 4.15, 6.38, and 14.76 μg/L, respectively.

Compared to WQCs in other studies, it could be clearly seen that the site-specific SWQCs and LWQCs of copper, cadmium, and lead in FR were higher than the national recommended values in several countries and those of other rivers in China (Table 5). The SWQC and LWQC of Cr were the lowest, while the SWQCs and LWQCs of zinc, manganese, and nickel were at a medium level. The differences might be caused by the selection of species, diversity of water quality parameters, and data processing (i.e., standardization of toxicity data). Previous studies had reported the differences in sensitivity between native and non-native species [29,55] Aquatic biota has obvious regional characteristics, which are not only reflected in the distribution differences of native species at home and abroad but also in species from different watersheds in China. Fen River, Tai Lake [49], Lancang River [56], Shaying River [40], and Liao River Basin [56] have different geographical and climatic conditions, which promote their unique biological communities and contribute to the differences in regional WQCs. In the US EPA guidelines, it was recommended that only toxicity data for species native to an area of interest be used to develop site-specific WQC [37]. The purpose of this recommendation was to minimize uncertainties associated with differences in natural history, aquatic system characteristics, taxonomic groups, habitats, and the geographical distribution of species [37]. The selected aquatic organisms in the derivation of site-specific WQCs must be species representing the characteristics of aquatic biota in each area, so as to obtain WQCs that could provide sufficient protection for most organisms. 

In addition, the bioavailability and toxicity of heavy metals depend on water chemical parameters [59]. DOC, hardness, and pH are the important water quality parameters affecting metal toxicity. DOC plays a key role in metal morphology and toxicity and can reduce metal toxicity through complexation [60,61]. The toxicity of heavy metals has been shown to decrease with an increase in water hardness and alkalinity [62]. Higher values of pH, DOC, alkalinity, and hardness were observed in FR compared to those in other water bodies. In particular, DOC (8.82 mg/L) was about 17 times greater than that of 0.5 mg/L in US water bodies [20], about 7 times higher than that of 1.12 mg/L measured in Lancang River [56], and about 2 times larger than that in Tai Lake (4.94 mg/L) [51]. The hardness of FR was also significantly higher than that of the United States (50 mg/L) [20], Australia (50 mg/L), Tai Lake (150 mg/L) [41], and Shaying River (100 mg/L) [40]. In this study, the toxicity data of native aquatic species in the Fen River Basin were selected and corrected according to the regional water quality parameters. The site-specific WQCs derived in the present study can provide better protection for native aquatic organisms against the toxic effects of corresponding metals.

### 3.3. Ecological Risk

**Figure 5:**
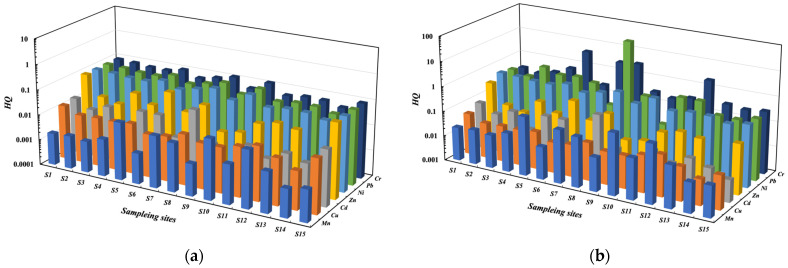
HQ values of metals in aquatic organisms at different sampling points ((**a**): acute hazard quotient, (**b**): chronic hazard quotient).

The corresponding HQ values in FR were obtained (Figure 5; Tables S11 and S12). The acute and chronic HQ values of Cd, Cu, Zn, Ni, and Mn at all sampling sites were lower than 1, indicating low environmental risk posed by these metals to native aquatic organisms in FR. The HQs of Pb at the sampling site, Duanchun River (S8), were the highest. The acute HQ value of Pb at the site of S8 exceeded 1 (HQ: 1.53), and the chronic HQ value was 39.4. It indicated that Pb posed a moderate to high hazardous risk to the aquatic organisms in FR.

The acute and chronic HQ values of Cr at four sampling sites (S5, S7, S8, and S12) were greater than 1, which indicated that Cr posed a high risk to aquatic organisms. The abnormal concentration of Cr at the site of S8 in FR was mainly due to some enterprises, such as coal mines, near the sampling areas. The sampling sites (S5 and S7) are located in Linfen City, in the south of Shanxi Province. As a resource-based city, Linfen City has many energy and chemical enterprises, most of which are distributed in Hongdong County, followed by Yaodu District. Most of these enterprises were heavy industries, such as coal coke, steel, and chemical industry, with serious pollution [63]. Therefore, the main pollution at the sites of Yaodu District (S5) and Hongdong Shitan (S7) would be caused by anthropogenic activities such as mineral resource development. The sampling sites 12 was located in Taiyuan, the capital city of Shanxi Province. There are many metal smelting enterprises, coal mine production bases, and other factories in the Taiyuan Section of FR. At least 885 million tons of sewage discharge were released from 51 sewage outlets annually [64]. Therefore, Cr at the site of the Xiao River (S12) in Taiyuan Xiaodian reaches would be related to industrial sewage discharge.

**Figure 6:**
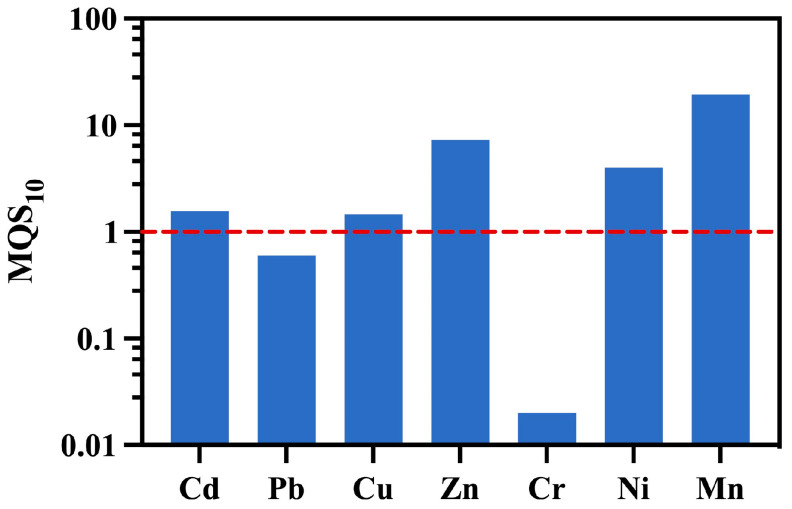
Margin of safety for seven metals in surface waters in the Fen River (The red dashed line is risk level 1).

The MOS_10_ values of Cd, Cu, Zn, Ni, and Mn were greater than 1 (Table 6 and Figure 6), which indicated that these elements posed low ecological risk to aquatic organisms in FR. The MOS_10_ of Pb and Cr was 0.60 and 0.02, respectively, and these two metals posed a high ecological risk to aquatic organisms in FR, especially Cr. It is necessary to monitor these two potential risk factors and take effective measures to reduce the input of anthropogenic factors in FR. According to the results of these two methods for ERA, Pb and Cr were the main ecological risk factors in FR.

The accuracy of WQCs can have an effect on ecological risk assessment, and it is particularly important to reduce the uncertainty in the derivation of WQCs. The site-specific WQCs were derived in this study and used in the risk assessment, which could provide appropriate and sufficient protection for aquatic life in the Fen River Basin. It must be noted that due to the lack of sufficient chronic toxicity data, the final ACRs were used to derive the LWQCs, resulting in some uncertainty in the WQCs. More toxicity data should be obtained to improve the accuracy of WQCs. In addition, relatively sparse sampling points may lead to uncertainty in the distribution of Cr and Pb. In the future, more detailed work should be carried out in these areas with high Cr and Pb contents.

## 4. Conclusions

The concentration and distribution characteristics of typical heavy metals were investigated in the Fen River in the wet season of 2020, and the ecological risk assessments of these metals were evaluated based on site-specific WQCs. There was pollution in various degrees at sites in the Yaodu District (S5), Shitan (S7), and Duanchun River (S8) taken from the middle-lower reaches. The concentrations of Pb in the water samples exceeded the water quality standard of class V. The single-factor pollution index for Cr and Pb and the integrated pollution index for Pb were the highest, indicating heavy pollution levels. The site-specific WQCs of seven metals were derived based on the water quality parameters and toxicity data for native aquatic species in FR. Based on the site-specific WQCs and tiered methods for risk assessment (HQ and MOS_10_), Pb and Cr were the most important pollutants and might have hazardous effects on the aquatic organisms in FR. Therefore, it is suggested to continuously carry out the monitoring and evaluation of heavy metal pollution in FR. In areas with high ecological risk of heavy metals, we should strengthen pollution prevention and reduce anthropogenic inputs of Pb and Cr from various industries.

## Figures and Tables

**Table 1:** Standards for single-factor pollution index (*P_i_*) and integrated pollution index (*I*).

*P_i_*	Pollution Grade	Pollution Level	*I*	Pollution Grade	Pollution Level
*P_i_* < 1	safety	clean	*I* < 1	safety	clean
1 ≤ *P_i_* < 2	alert levels	cleanliness	1 ≤ *I* < 2	alert levels	light pollution
2 ≤ *P_i_* < 3	mild pollution	pollution	2 ≤ *I* < 3	mild pollution	pollution
3 ≤ *P_i_*	heavy pollution	heavy pollution	3 ≤ *I* < 5	heavy pollution	heavy pollution
			5 ≤ *I*	severe pollution	severe pollution

**Table 2:** Distribution characteristics of heavy metals in the Fen River (μg/L).

Metal	Cd	Pb	Cu	Zn	Cr	Ni	Mn
Con. range	0.0032–0.5007	0.2071–251.18	0.3177–1.357	0.0766–5.718	0.8873–17.12	4.341–12.18	2.599–47.72
Mean	0.12	19.06	0.79	1.82	4.22	7.51	13.73
SD ^a^	0.16	64.23	0.29	1.61	5.32	2.35	15.21
CV% ^b^	107.19	337.04	36.61	88.84	126.10	31.34	110.82
CSS (III) ^c^	5	5	1000	1000	50	20	100
Overshooting rates (%)	0	6.67	0	0	0	0	0
Water Quality	Ⅰ	Ⅰ–Ⅴ	Ⅰ	Ⅰ	Ⅰ–Ⅱ	Ⅰ	Ⅰ
Upstream	0.017	1.83	0.66	1.94	1.30	5.75	2.92
Midstream	0.10 ± 0.18	37.57 ± 94.20	0.78 ± 0.28	1.22 ± 0.99	3.84 ± 4.73	7.35 ± 3.25	17.54 ± 17.79
Downstream	0.11 ± 0.06	3.01 ± 1.66	0.82 ± 0.33	2.39 ± 2.07	5.01 ± 6.42	7.92 ± 1.28	11.45 ± 13.45

Note: ^a^ standard deviation; ^b^ coefficient of variation; ^c^ standard was from environment quality standards for surface water (III) (State Environmental Protection Administration, 2002).

**Table 3:** Comparison of heavy metal concentrations in the Fen River and other rivers (μg/L).

Rivers	Sampling Time	Cd	Pb	Cu	Zn	Cr	Ni	Mn	Reference
Fen River, China	August 2005	1.74	7.48	13.12	54.50	32.20	-	498.18	[12]
Fen River, China	September 2015	-	-	-	-	-	8.20	-	[15]
Fen River, China	May 2019	-	-	2.40	-	-	5.87	67.96	[11]
Fen River, China	September 2019	-	-	2.46	-	-	1.50	64.08	[11]
Fen River, China	September 2020	0.12	19.20	0.79	1.81	4.24	7.52	13.82	This study
Chao Lake, China	August 2020	5.08	1.34	121.75	89.38	0.48	2.62	22.89	[35]
Shaying River, China	July 2018	0.14	0.96	1.03	9.03	0.38	0.95	56.82	[44]
Tai Lake, China	January 2016	0.93	45.88	65.24	185.64	-	-	-	[45]
Huang River, China	August 2005	0.29	3.10	5.60	7.30	3.05	-	118.0	[12]
Dongting Lake, China	July–August 2007	0.05	1.49	2.50	20.91	0.62	1.14	-	[46]
Jiulongjiang River, China	April 2013	0.08	4.47	17.85	154.89	5.41	3.99	-	[47]
Daye River, China	2013–2014	1.3	0.96	1.6	4.7	1.6	-	-	[48]
World average	-	0.08	0.079	1.48	0.60	0.70	0.80	34.0	[49]

**Table 6:** Margin of safety for seven metals in surface waters in the Fen River.

Parameter	Cd	Pb	Cu	Zn	Cr(Ⅵ)	Ni	Mn
SSD_10_	0.36	8.6	1.75	2.68	0.13	42.26	527.13
ECD_90_	0.23	14.23	1.20	0.37	6.94	10.58	27.23
MQS_10_	1.57	0.60	1.46	7.24	0.02	3.99	19.36

**Table 4:** Parameters of the fitting model and WQCs of metals to freshwater aquatic organisms (μg/L).

Metal	Type	Model	n	HC_5_^e^	R^2 f^	RMSE ^g^	KSp ^h^	WQC	Method
Mn ^a^	SWQC ^c^	Log-normal	20	4052.29	0.9759	0.0426	0.9879	2026.15	Hardness-SSD
	LWQC ^d^	Logistic	11	333.05	0.8528	0.1011	0.6685	166.53	Invertebrates and fish, Hardness-SSD; Plant, pH-SSD
Cu	SWQC	Log-logistic	11	197.24	0.9649	0.0493	0.9999	98.62	BLM-SSD
	LWQC							29.71	ACR ^b^ (6.64) [51]
Cd	SWQC	Log-logistic	19	20.03	0.9450	0.0642	0.6278	10.02	Hardness-SSD
	LWQC							2.18	ACR ^b^ (9.18) [40]
Zn	SWQC	Normal	17	126.14	0.9227	0.0757	0.6201	63.07	Hardness-SSD
	LWQC							19.29	ACR ^b^ (6.54) [52]
Cr	SWQC	Log-logistic	19	12.11	0.9710	0.0467	0.8756	6.06	SSD
	LWQC							4.15	ACR ^b^ (2.917) [53]
Pb	SWQC	Log-normal	15	327.47	0.9323	0.0486	0.8966	163.74	Hardness-SSD
	LWQC							6.38	ACR ^b^ (51.29) [53]
Ni	SWQC	Logistic	18	265.46	0.9412	0.0662	0.8905	132.73	Hardness-SSD
	LWQC							14.76	ACR ^b^ (17.99) [54]

Note: ^a^ Unit (mg/L); ^b^ ACR is the acute-to-chronic ratio; ^c^ SWQC is the short-term WQC; ^d^ LWQC is the long-term WQC; ^e^ HC_5_ is hazardous concentration 5; ^f^ R^2^ is the coefficient of determination; ^g^ RMSE is the root mean square errors; ^h^ KSp is the K-S test.

**Table 5:** WQCs of seven metals in various water bodies at home and abroad.

Metal	Region and Reference	Method	WQC/(μg/L)	
			SWQC ^e^	LWQC ^f^
Cu	Fen River (This study)	BLM-SSD	98.62	29.71
	Tai Lake [51]	BLM-SSD	53.50	16.10
	Lancang River [56]	BLM-SSD	26.79	1.11
	US EPA [20]	BLM-TPR	2.34	1.45
	Canada ^b^	Hardness-SSD	-	3.91
	Australia ^c^	Hardness-SSD	-	1.4
Zn	Fen River (This study)	Hardness-SSD	63.07	19.29
	Tai Lake [41]	Hardness-SSD	100.69	30.79
	US EPA ^d^	Hardness-TPR ^g^	120	120
	Australia ^c^	SSD	8	8
Cd	Fen River (This study)	Hardness-SSD	10.02	2.18
	Shaying River [40]	Hardness-TPR	6.46	1.49
	China [57]	Hardness-SSD	6.5	0.29
	US EPA ^d^	Hardness-TPR	1.8	0.72
	Australia ^c^	SSD	-	0.2
	Canada ^b^	Hardness-SSD	3.8	0.26
Cr	Fen River (This study)	SSD	6.06	4.65
	Tai Lake [57]	TPR	20.42	5.44
	Liao River [57]	TPR	16.34	4.45
	US EPA ^d^	TPR	16	11
Mn ^a^	Fen River (This study)	Hardness, pH-SSD	2026	166
	Canada ^b^	Hardness, pH-SSD	3600	430
	Australia ^c^	SSD	-	1900
Pb	Fen River (This study)	Hardness-SSD	163.74	6.38
	Tai Lake [58]	Hardness-SSD	122.45	4.77
	US EPA ^d^	Hardness-TPR	65	2.5
	Canada ^b^	Hardness-SSD	-	6.72
Ni	Fen River (This study)	Hardness-SSD	132.73	18.65
	US EPA ^d^	Hardness-TPR	470	52
	Canada ^b^	Hardness-SSD	-	149

Note: ^a^ Unit (mg/L); ^b^ https://ccme.ca/en/summary-table (accessed on 24 February 2023); ^c^ https://www.waterquaity.gov.au/anz-guidelines/guideline-values/default/water-quality-toxicants/search (accessed on 15 February 2023); ^d^ https://www.epa.gov/wqc/national-recommended-water-quality-criteria-aquatic-life-criteria-table#table (accessed on 21 January 2023); ^e^ SWQC is the short-term WQC; ^f^ LWQC is the long-term WQC; ^g^ TPR is the toxicity percent ranked method.

## Data Availability

Not applicable.

## Supplementary Materials

The following supporting information can be downloaded at: https://www.mdpi.com/article/10.3390/toxics11080704/s1, Table S1: Acute toxicity of freshwater aquatic organisms of copper in Fen River; Table S2: Acute toxicity of freshwater aquatic organisms of lead in Fen River; Table S3: Acute toxicity of freshwater aquatic organisms of zinc in Fen River; Table S4: Acute toxicity of freshwater aquatic organisms of cadmium in Fen River; Table S5: Acute toxicity of freshwater aquatic organisms of chromium in Fen River; Table S6: Acute toxicity of freshwater aquatic organisms of nickel in Fen River; Table S7: Acute toxicity of freshwater aquatic organisms of manganese in Fen River; Table S8: Chronic toxicity of freshwater aquatic organisms of manganese in Fen River; Table S9: Water quality parameters in Fen River; Table S10: Acute hazard quotient of metals to aquatic organisms at different sampling points; Table S11: Chronic hazard quotient of metals to aquatic organisms at different sampling points; Table S12: Pollution index of heavy metals in Fen River. References [65,66,67,68,69,70,71,72,73,74,75,76,77,78,79,80,81,82,83,84,85,86,87,88,89,90,91,92,93,94,95,96,97,98,99,100,101,102,103,104,105,106,107,108,109,110,111,112,113,114,115,116,117,118,119,120,121,122,123,124,125,126,127,128,129,130,131,132,133,134,135,136,137,138,139,140,141,142,143,144,145,146,147,148,149,150,151,152,153,154,155,156,157,158,159,160,161,162,163,164,165,166,167,168,169,170,171,172,173,174,175] are cited in the supplementary materials.
