# Supplementary material for: Distribution, Site-Specific Water Quality Criteria, and Ecological Risk Assessment of Heavy Metals in Surface Water in Fen River, China
Source: Toxics. 2023 Aug 15;11(8):704. doi: 10.3390/toxics11080704 (PMC10458924; doi:10.3390/toxics11080704)
Supplement: Supplementary file 1 [file toxics-11-00704-s001.zip › toxics-2532195-supplementary.pdf]

Table S1 Acute toxicity of freshwater aquatic organisms of copper in Fen River

Species	Total copper LC_{50}μg/L	Dissolved Copper/μg/L	Hardness $CaCO_3$/mgL	Tem[1]℃	pH	DOC mg/L	Ca mg/L	Mg mg/L	Na mg/L	K mg/L	SO_4^{2-} mg/L	Cl^- mg/L	Alkalinity mg/L	Reference
Lumbriculus variegatus	130	—	290	25	6.57	0.5	47.8602	41.47	89.821	7.178	278.4	6.508	235	[1]
	270	—	290	25	7.29	0.5	47.8602	41.47	89.821	7.178	278.4	6.508	235	
	500	—	290	25	5.25	0.5	47.8602	41.47	89.821	7.178	278.4	6.508	235	
Ceriodaphnia dubia	19	—	52	24.5	7.5	1.1	15.2833	3.3713	1.5	0.57	3.8	1.4	55	[2]
	17	—	52	24.5	7.5	1.1	15.2833	3.3713	1.5	0.57	3.8	1.4	55	[3]
	—	25	45	25	7.72	1.5	11.0991	4.2075	9.5	1.6	46	34	39.7	
	—	17	45	25	7.72	1.5	11.0991	4.2075	9.5	1.6	46	34	39.7	
	—	30	45	25	7.72	1.5	11.0991	4.2075	9.5	1.6	46	34	39.7	
	—	24	45	25	7.72	1.5	11.0991	4.2075	9.5	1.6	46	34	39.7	
	—	28	45	25	7.72	1.5	11.0991	4.2075	9.5	1.6	46	34	39.7	
	—	32	45	25	7.72	1.5	11.0991	4.2075	9.5	1.6	46	34	39.7	
	—	23	45	25	7.72	1.5	11.0991	4.2075	9.5	1.6	46	34	39.7	
	—	20	45	25	7.72	1.5	11.0991	4.2075	9.5	1.6	46	34	39.7	

—	19	45	25	7.72	1.5	11.0991	4.2075	9.5	1.6	46	34	39.7	
—	26	94.1	25	8.15	2	23.2094	8.7984	5.2449	1.6	20.054	6.171	69.6	
—	21	94.1	25	8.15	2	23.2094	8.7984	5.2449	1.6	20.054	6.171	69.6	
—	27	94.1	25	8.15	2	23.2094	8.7984	5.2449	1.6	20.054	6.171	69.6	
—	37	94.1	25	8.15	2	23.2094	8.7984	5.2449	1.6	20.054	6.171	69.6	
—	34	94.1	25	8.15	2	23.2094	8.7984	5.2449	1.6	20.054	6.171	69.6	
—	67	179	25	8.31	2.3	50.1069	13.1232	14.32	2.4	22.673	10.979	140.1	
—	38	179	25	8.31	2.3	50.1069	13.1232	14.32	2.4	22.673	10.979	140.1	
—	78	179	25	8.31	2.3	50.1069	13.1232	14.32	2.4	22.673	10.979	140.1	
—	81	179	25	8.31	2.3	50.1069	13.1232	14.32	2.4	22.673	10.979	140.1	
—	28	97.6	25	8	2	24.0727	9.1256	5.44	1.6	20.8	6.4	74.2	[3]
—	84	182	25	8	2.3	50.9467	13.3432	14.56	2.4	23.053	11.163	144.3	
13.4	—	57.1	25	8.18	0.5	9.42352	8.1653	17.685	1.413	54.815	1.2814	81	[4]
6.98	—	80	20	7.6	0.5	13.2028	11.44	24.778	1.980	76.799	1.7953	53	[5]
25	24	105	25	8.4	0.5	26	8.5	8.7	1	18	9.1	100	[6]
111	106.56	102	25	8.3	2.5	25	8.3	8.5	0.9	16	9.4	91	

	157	150.72	106	25	8.3	5	26	8.5	9.2	1	20	11	94	
	147	141.12	103	25	8.3	5	26	8.5	9.2	1	20	11	93	
	267	256.32	106	25	8.3	10	26	8.6	9.4	1.1	29	16	96	
Daphnia magna	9.1	—	39	20	7.8	1.1	10.9867	2.7776	5.8136	0.7	7.9394	7.7684	51	[7]
	11.7	—	39	20	7.8	1.1	10.9867	2.7776	5.8136	0.7	7.9394	7.7684	51	
	6.6	—	39	20	7.79	1.1	10.7129	2.7203	5.7423	0.7	7.6578	7.6406	50	
	9.9	—	39	20	7.79	1.1	10.7129	2.7203	5.7423	0.7	7.6578	7.6406	50	
	11.7	—	39	20	6.9	1.1	10.9867	2.7776	5.8136	0.7	7.9394	7.7684	30	
	6.7	—	39	20	6.9	1.1	10.9867	2.7776	5.8136	0.7	7.9394	7.7684	30	
	9.1	—	26	20	7.6	1.1	7.4273	2.0327	4.8867	0.7	4.2786	6.107	24	
	5.2	—	27	20	7.7	1.1	7.7011	2.09	4.958	0.7	4.5602	6.2348	24	
	41.2	—	170	20	7.8	0.5	27.9433	24.2353	52.507	4.196	162.74	3.8045	115	[8]
	10.5	—	170	20	7.8	0.5	27.9433	24.2353	52.507	4.196	162.74	3.8045	115	
	20.6	—	170	20	7.8	0.5	27.9433	24.2353	52.507	4.196	162.74	3.8045	115	
	17.3	—	170	20	7.8	0.5	27.9433	24.2353	52.507	4.1961	162.74	3.8045	115	
	70.7	—	170	20	7.8	0.5	27.9433	24.2353	52.507	4.1961	162.74	3.8045	115	

31.3	—	170	20	7.8	0.5	27.9433	24.2353	52.507	4.1961	162.74	3.8045	115	
7.1	—	109.9	21	6.93	2.4	40	2.43	85.1	1.23	10	106	12.5	
16.4	—	109.9	21	6.93	3.4	40	2.43	85.1	1.23	10	106	12.5	
39.9	—	109.9	21	7.43	3.4	40	2.43	85.1	1.23	10	106	13.875	
18.7	—	109.9	21	7.43	2.4	40	2.43	85.1	1.23	10	106	13.875	
18.9	—	109.9	21	7.82	2.4	40	2.43	85.1	1.23	10	106	14.5	[9]
39.7	—	109.9	21	7.82	3.4	40	2.43	85.1	1.23	10	106	14.5	
46	—	109.9	21	6.93	4.4	40	2.43	85.1	1.23	10	106	12.5	
71.9	—	109.9	21	6.93	6.1	40	2.43	85.1	1.23	10	106	12.5	
57.2	—	109.9	21	7.43	4.4	40	2.43	85.1	1.23	10	106	13.875	
67.8	—	109.9	21	7.82	4.4	40	2.43	85.1	1.23	10	106	14.5	
26	—	52	18.2	7.8	1.1	14	3.5	12	2.9	23	11	45	
30	—	105	20.3	7.9	1.1	29	6.8	29	5.3	57	21	79	
38	—	106	19.7	8.1	1.1	29	6.8	29	5.3	57	21	82	[10]
69	—	207	19.9	8.3	1.1	58	13	62	8.2	127	40	166	
4.8	—	7.1	24	8.55	0.5	1.15182	1.02739	3.5102	2.8052	6.8159	2.5434	56	

	7.4	—	20.6	24	6.97	0.5	3.39973	2.9458	2.5478	2.1356	19.776	1.9363	60	
	6.5	—	23	24	8.52	0.5	3.79581	3.289	2.8446	2.3845	22.08	2.1619	64	
	197.53	—	198.6471	21	7.57	11.12	77.13	1.47	50.47	0.46	55	33	142	
	134.55	—	903.6216	21	7.98	4.99	66.35	179.2	35.31	0.51	76	43.8	193	
	244.45	—	167.8175	21	8	20.12	64.47	1.66	126.7	0.47	52	57.6	219	
	155.69	—	997.6020	21	8.07	10.84	78.59	194.6	64.93	0.53	90	72.4	258	
	350	—	120.3696	21	7.98	11.95	46.54	1.01	70.01	0.59	63	50.8	69	[11]
	210.49	—	417.1427	21	7.72	7.31	104.9	37.69	128.4	7.31	67	66.2	87	
	194.78	—	136.5252	21	7.56	10.06	53.01	1.01	58.54	0.6	55	44	79	
	187.77	—	457.7211	21	7.86	6.44	111.8	43.36	140.9	15.46	66	54	98	
	71.46	—	12.047	21	8.4	1.9	4	0.5	0.75	0.35	0.1	2	48	
	365.7	351.072	156.3014	21	8.1	0.05	44.9	10.73	69.5	10	42.92	88.8	138.58	
	425.48	408.4608	156.3014	21	8.3	1	44.9	10.73	69.5	10	42.92	88.8	138.58	
Hypophthalmichtys molitrix	270.04	259.2384	156.3014	21	8.3	0.5	44.9	10.73	69.5	10	42.92	88.8	138.58	[12]
	639.77	614.1792	156.3014	21	8.3	2	44.9	10.73	69.5	10	42.92	88.8	138.58	
	1138.78	1093.2288	156.3014	21	8.3	4	44.9	10.73	69.5	10	42.92	88.8	138.58	

Ctenopharyngodon idellus	2364.09	2269.5264	156.3014	22	7.9	4	44.9	10.73	70.65	10	42.92	88.8	138.58	[12]
Ctenopharyngodon idellus	917.89	881.1744	156.3014	22.1	8	2	44.9	10.73	61.45	10	42.92	88.8	138.58	[12]
	846.06	812.2176	156.3014	22.3	7.9	0.5	44.9	10.73	56.85	10	42.92	88.8	138.58	
	772.68	741.7728	156.3014	21	7.9	1	44.9	10.73	54.55	10	42.92	88.8	138.58	
	831.19	797.9424	156.3014	21	7.9	0.05	44.9	10.73	52.25	10	42.92	88.8	138.58	
Ctenophryodon idellus	846.67	812.8032	—	20	8	1	44.9	10.73	69.5	10	42.92	88.8	138.58	[13]
	773.24	742.3104	—	20	8	2	44.9	10.73	69.5	10	42.92	88.8	138.58	
	918.56	881.8176	—	20	8	3	44.9	10.73	69.5	10	42.92	88.8	138.58	
	2365.8	2271.168	—	20	8	4	44.9	10.73	69.5	10	42.92	88.8	138.58	
Macrobrachium nipponense	417	—	253	18.6	7.78	0.05	80.1818	12.1694	17.7074	3.0158	48.069	73.6497	44	[14]
Cyprinus carpio	912	—	248	18.5	7.84	0.05	80.1818	12.1694	17.7074	3.0158	48.069	73.6497	44	[14]
Pseudorasbora parva	347	—	238	18.4	7.73	0.05	80.1818	12.1694	17.7074	3.0158	48.069	73.6497	44	[14]

Carassius auratus	1405	—	248	18.5	7.85	0.05	80.1818	12.1694	17.7074	3.0158	48.069	73.6497	44	[14]
Misgurnus anguillicaudatus	24	—	256	19.1	7.81	0.05	80.1818	12.1694	17.7074	3.0158	48.069	73.6497	44	[14]
Chironomus plumosus	10^6	—	251	18.5	7.74	0.05	80.1818	12.1694	17.7074	3.0158	48.069	73.6497	44	[14]

Note: [1] Temp. is temperature.

Table S2 Acute toxicity of freshwater aquatic organisms of lead in Fen River

Species	ATV[1](ug/L)	Hardness ($CaCO_3$ mg/L)	Reference
*Daphnia magna**	3610	240	[15]
	894	150	[16]
	450	45	[17]
	1910	152	[18]
Tubifex tubifex	165220	237	[19]
	334140	237	
	454760	237	
	514190	237	
	450000	224	[20]
*Ceriodaphnia dubia**	2700	290	[1]
	248	100	[21]
	208.8	82.4	[22]
	256.9	80	[23]
	1760.4	172	
*Cyprinus carpio**	1190	120	[24]
	1350	58	
	1780	90	
	1890	170	
	3530	120	
	1400	280	
	13350	360	[25]
*Chironomus tentans**	34670	25	[26]
	220000	224	[20]
*Lumbriculus variegatus**	8000	290	[27]
	1800	30	
*Ctenopharyngodon idllus**	576410	160	[28]
	3730	10	[29]
*Carassius auratus**	273900	160	[30]
	11590	10	[29]
	169470	120	[31]
Rhodeus ocellatus	13187	250	[32]
Daphnia carinata	444	82.4	[22]
Tanytarsus dissimilis	258	46.8	[33]
Chlofella saccharophila	63800	-	[34]
*Rhodeus ocellatus**	6784	50	[35]
	25557	150	
	44325	250	
*Margarga melanoiides**	3052	50	[35]
	22457	150	

	39644	250	
*Pseudorasbora parva**	9155	50	[35]
	38950	150	
	60216	250	
*Misgurnus anguillicaudatus**	12772	50	[35]
	42873	150	
	63838	250	

Note: [1] ATV is acute toxicity value; All species used of water quality reference normalization were marked with an *.

Table S3 Acute toxicity of freshwater aquatic organisms of zinc in Fen River

**Species**	**ATV[1](μg/L)**	**Hardness ($CaCO_3$ mg/L)**	**Reference**
Cyprinus carpio	7800	55	[36]
	3120	19	[37]
Daphnia sp.*	500	6.5	[38]
	5200	400	
	100	45	[17]
Chironomus sp.*	890	50	[39]
	1160	100	
	1630	250	
	2590	400	
*Daphnia magna**	90	50	[39]
	140	100	
	230	250	
	690	400	
*Limnodrilus hoffmeistteri**	1950	50	[39]
	2290	100	
	3330	250	
	6310	400	
*Cipangopaludian cathayensis**	2340	50	[39]
	3070	100	
	4760	250	
	7690	400	
*Pseudorasbora parva**	2570	150	[40]
	18000	250	[39]
	20600	400	
Gyraulus sp.	3292	62	[41]
	1400	14	
	1451	18	
Gammarus sp.	8100	50	[36]
Ceriodaphnia dubia	180	52	[42]
Lemna minor	3014	100	[43]

Chlorella vulgaris	153	100	[44]
Ceriodaphnia dubia	354	280	[45]
Misgurnus anguillicaudatus	16910	150	[40]
Ctenopharyngodon idellus	31370	120	[46]
Rhodeus ocellatus	8004	150	[40]
Margarga melanoiides	7945	150	[40]
Palaemon modestus	267	150	[40]

Note: [1] ATV is acute toxicity value; All species used of water quality reference normalization were marked with an *.

Table S4 Acute toxicity of freshwater aquatic organisms of cadmium in Fen River

Species	ATV[1](ug/L)	Hardness ($CaCO_3$ mg/L)	Reference
*Carassius auratus**	2193	50	[47]
	11123	150	
	13938	250	
*Limnodrilus hoffmeistteri**	188	50	
	227	153	
	431	265	
*Pelteobagrus fulvidraco**	3909	50	
	13609	150	
	17397	250	
*Daphnia pulex**	47	57	[48]
	319	240	[49]
	100	120	[50]
*Cyprinus carpio**	17100	100	[51]
	240	55	[36]
	220770	185	[52]
*Tubifex tubifex**	61470	237	[19]
	440	45	
	7950	173	
	8500	305	
	1658	250	
*Lumbriculus variegates**	120	30	[27]
	780	290	[1]
*Ceriodaphnia dubia**	31.47	40	[53]
	361.1	172	[23]
Diaphanosoma brachyurum	1060	93	[54]
Moina macrocopa	71.25	82	[55]
Gammarus pseudolimnaeu	49	76.8	[56]
Lemna minor	54	39	[57]
Gammarus pulex	50	117.4	[58]

Daphnia magna	3.6	170	[8]
*Palaemon modestus**	6	50	[59]
	12	150	
	18	250	
*Misgurnus anguillicaudatus**	3054	50	
	11647	150	
	18849	250	
*Pseudorasbora parva**	3318	50	
	5512	150	
	8503	250	
*Margarga melanoiides**	1868	50	
	3969	150	
	5659	250	
*Rhodeus ocellatus**	2825	50	
	7833	150	
	10072	250	

Note: [1] ATV is acute toxicity value; All species used of water quality reference normalization were marked with an *.

Table S5 Acute toxicity of freshwater aquatic organisms of chromium in Fen River

Species	ATV[1](ug/L)	Hardness ($CaCO_3$ mg/L)	Reference
Daphnia magna	24.2	45	[60]
	66.7	188	[61]
	73.6	196	
	75.8	213	
	85.7	196	
	131	185	
	137	212	
	157	92	[62]
	164	185	[61]
	175	100	[62]
	212	213	[61]
	250	93	[63]
	550	180	[64]
	1300	-	[65]
	778	-	[66]
	19.9	50	[61]
	21.3	50	
	157	212	
	15.3	50	
	20.6	50	
	22	45	[67]

Daphnia pulex	36.3	45	[68]
	48	45	[67]
	220	50	[69]
	260	171	[70]
Lymnaea Luteola	3880	195	[71]
Tubifex tubifex	872	237	[19]
	1227	237	
	1846	237	
	2720	237	
	9800	123	[72]
	190	245	[73]
Lumbriculus variegatus	11000	130	[74]
	13300	30	[27]
Ceriodaphnia sp.	144	100	[21]
	1500	102	[75]
	45.2	45	[60]
	45	45	[67]
Carassius auratus	168500	120	[76]
	94570	150	[77]
	244100	300	[78]
	37500	20	[79]
Cyprinus carpio	123000	220	[80]
Macrobrachium nipponensis	2241	-	[81]
	38.48	120	[76]
Moina macrocopa	360	-	[82]
Carassius auratus	139000	-	[83]
Hypophthalmichthys molitrix	13160	120	[76]
Scenedesmus obliquus	680	-	[84]
Scenedesmus quadricauda	20890	-	[85]
Pseudokirchneriella subcapitata	129.7	-	[86]
	84.3	-	
Pelteobagrus fulvidraco	15790	120	[87]
	57980	120	
Margarga melanoiides	3.602	150	[35]
Pseudorasbora parva	30.341	150	
Misgurnus anguillicaudatus	35.491	150	
Palaemon modestus	0.357	150	

Note: [1] ATV is acute toxicity value.

Table S6 Acute toxicity of freshwater aquatic organisms of nickel in Fen River

Species	ATV[1](ug/L)	Hardness ($CaCO_3$mg/L)	Reference
*Ceriodaphnia dubia**	148	113	[88]
	400	253	
	81	50	
	137.37	172	[23]
	64.80	80	
	674	142	[89]
	30	69.5	[90]
Desmodesmus subspicatus	360	-	[91]
Lemna minor	450	-	[92]
	210	-	[93]
	360	-	
	330	-	
	1290	-	[94]
Scenedesmus quadricauda	1500	-	[91]
	900	-	[95]
*Hyalella azteca**	393.07	240	[96]
	3045	98	[88]
	2000	119.5	[97]
Daphnia pulex	1480	142	[89]
	3890	142	
	912.33	127	[98]
*Daphnia magna**	1800	51	[18]
	2360	100	
	1920	104	
	4970	206	
	7300	240	[99]
	7590	240	
	915	51.1	[100]
*Danio rerio**	9068	141	[101]
	18919	141	
	13120	141	
	589.9	7.8	
Tubifex tubifex	25100	237	[19]
	18000	237	
	18970	237	
	25460	237	
*Oncorhynchus mykiss**	19200	250	[83]
	8900	33	[102]
	7790	50	[103]

	15000	285	[104]
	13700	35	[105]
	16300	200	
Carassius auratus	63000	126	[106]
Chironomus sp.	8.6	50	[107]
Lumbriculus variegatus	32000	130	[74]
	48000	130	
*Cyprinus carpio**	10400	55	[36]
	40800	126	[106]
	43650	305	[108]
	14790	305	
	48000	305	
	6900	305	
	35000	112	[109]
	6160	128	[110]
	10600	53	[111]
Gammarus fasciatus	100000	130	[74]
Chironomus dilutus	119500	119.5	[97]
Paramisgurnus dabryanus	228560	150	[112]
Misgurnus anguillicaudatus	275420	150	[112]

Note: [1] ATV is acute toxicity value; All species used of water quality reference normalization were marked with an *.

Table S7 Acute toxicity of freshwater aquatic organisms of manganese in Fen River

**Species**	**ATV[1](ug/L)**	**Hardness ($CaCO_3$mg/L)**	**Reference**
Nais elinguis	360	18.72	[113]
Desmodesmus subspicatus	15300	-	[114]
	12100	-	
	22800	-	
	8030	-	
	5920	-	
Closterium lunula	12600	-	[115]
*Hyalella azteca**	8567	80	[116]
	3000	26	
	13700	164	
*Daphnia magna**	10280	170	
	19400	170	
	9800	45.3	
Chlorella pyrenoidosa	17000	-	[115]
*Ceriodaphnia dubia**	14500	92	[116]
	14500	92	

	6700	26	
	5700	26	
	15900	184	
	14500	184	
Chironomus javanus	5270	18.72	[113]
*Chironomus tentans**	5800	25	[116]
	42200	100	
	94300	250	
Macrobrachium lanchesteri	9290	18.72	[113]
Lampsilis siliquoidea	43300	90	[116]
Aeolosoma sp.	39460	52	
Limnodrilus hoffmeisteri	239390	237	[19]
	239270	237	
	275700	237	
	164550	237	
*Tubifex tubifex**	26800	12	[116]
	42700	45	
	85900	173	
	464750	305	
Lymnaea stagnales	255530	172	
	205250	184	
Ctenopharyngodon idellus	541070	104.9	[117]
Cyprinus carpio	577270	104.9	
Asellus aquaticus	333000	50	[116]
Crangonyx pseudogracilis	694000	50	
Danio rerio	8280000	86.6	[118]

Note: [1] ATV is acute toxicity value; All species used of water quality reference normalization were marked with an *.

Table S8 Chronic toxicity of freshwater aquatic organisms of manganese in Fen River

Species	CTV[1](ug/L)	Hardess ($CaCO_3$ mg/L)	Reference
Danio rerio	4629.1	95	[116]
*Daphnia magna**	5400	100	
	9400	250	
*Oncorhynchus mykiss**	920	29.2	
	2182	151.2	
	700	50	
Aeolosoma sp.	2520	48	
*Ceriodaphnia dubia**	1045.1	60	
	1355.6	48	
	1364	52	

	2015	52	
	2241	24	
	2246	44	
	2498	20	
	2601	84	
	2826	720	
	2946	52	
	2962	52	
	2984	48	
	3100	76	
	3120	52	
	3250	52	
	3700	72	
	3858	44	
	3935	48	
	3956	52	
	4172	52	
	4782	400	
	4818	52	
	4846	52	
	5235	116	
	5554	200	
	6458	124	
	6526	48	
	7175	52	
	7484	304	
	7848	230	
	7962	384	
	8406	212	
	8439	284	
	8961	390	
	11235	410	
	14718	290	
Chironomus tentans	16340	89	
Hyalella azteca	382.8	104	
Lymnaea stagnalis	7700	174	
Lemna minor	23370	7.2	
Pseudokirchneriella subcapitata	123.00	8.3	
	173.00	8.1	
	194.40	7.9	
	273.00	7.8	

	327.00	8.4	
	333.00	7.85	
	362.00	7.75	
	448.50	8.15	
	597.00	7.9	
	621.00	7.95	
	622.70	7.8	
	623.00	7.8	
	634.60	7.65	
	2048.00	7.55	
	2455.00	6.95	
	4567.00	6.9	
	20063.00	6.05	
	36690.00	5.8	
Scenedesmus quadricauda	1910	7.5	

Note: [1] CTV is chronic toxicity value; All species used of water quality reference normalization were marked with an *.

Table S9 Water quality parameters in Fen River

Sample	Temp℃	pH	Hardness(mg/L $CaCO_3$)	DOC(mg C/L)	Ca^{2+} (mg/L)	Mg^{2+}(mg/L)	Na^+(mg/L)	K^+(mg/L)	SO_4^{2-} (mg/L)	Cl^- (mg/L)	Alkalinity(mg/L)
S1	23.8	8.48	320.29	10.11	81.94	24.93	75.94	7.39	342.17	105.10	327.60
S2	24.0	8.37	240.22	10.55	79.78	25.23	69.36	6.92	312.18	96.27	262.08
S3	23.3	8.28	220.20	10.87	67.95	24.29	86.29	8.36	274.04	110.92	393.12
S4	24.3	8.37	225.20	11.16	75.82	25.12	70.83	8.44	287.05	102.63	277.20
S5	23.9	8.07	270.24	9.08	68.21	25.18	62.63	7.97	260.97	85.94	352.80
S6	21.2	8.15	170.15	7.85	54.42	24.63	46.84	6.06	153.33	25.86	352.80
S7	24.1	8.15	159.14	10.61	59.77	25.16	66.89	7.54	228.52	82.73	252.00
S8	19.2	8.11	185.17	8.82	56.94	24.43	66.94	6.34	324.00	156.91	246.96
S9	22.0	8.13	179.16	8.12	54.27	24.26	51.55	5.29	169.80	64.34	252.00
S10	21.2	8.16	440.40	8.53	166.31	19.83	111.29	33.84	605.04	361.39	201.60
S11	20.9	8.35	120.11	12.17	33.44	25.09	126.21	17.03	201.73	177.37	252.00
S12	20.9	8.33	141.13	7.67	50.57	24.75	73.59	5.29	230.30	93.47	378.00
S13	20.7	8.34	180.16	8.16	34.16	24.08	57.06	11.32	121.58	74.62	267.12
S14	21.2	9.05	160.14	6.35	40.64	24.43	53.49	2.21	180.22	62.52	252.00
S15	21.2	8.31	200.18	8.20	66.36	24.66	49.13	5.48	93.00	46.29	443.52
Median value	21.2	8.31	182.66	8.82	59.77	24.66	40.34	7.39	230.30	93.47	267.12

Table S10 Acute hazard quotient of metals to aquatic organisms at different sampling points

Sample	HQ_{Acute}						
	Cd	Pb	Cu	Zn	Cr(VI)	Ni	Mn
S1	0.0159	0.0155	0.0137	0.0907	0.1988	0.0884	0.0018
S2	0.0076	0.0108	0.0078	0.0148	0.1538	0.0830	0.0020
S3	0.0134	0.0347	0.0083	0.0103	0.2264	0.0726	0.0017
S4	0.0034	0.0217	0.0083	0.0380	0.4305	0.0718	0.0029
S5	0.0164	0.0245	0.0095	0.0176	2.8249	0.0992	0.0186
S6	0.0165	0.0184	0.0050	0.0769	0.1552	0.0596	0.0016
S7	0.0029	0.0030	0.0058	0.0172	1.7929	0.0825	0.0109
S8	0.0500	1.5340	0.0098	0.0442	2.0534	0.1189	0.0082
S9	0.0032	0.0132	0.0062	0.0053	0.1997	0.0550	0.0018
S10	0.0003	0.0013	0.0061	0.0070	0.1464	0.1225	0.0236
S11	0.0054	0.0212	0.0092	0.0217	0.2035	0.0520	0.0036
S12	0.0024	0.0212	0.0138	0.0314	1.4401	0.0633	0.0182
S13	0.0054	0.0077	0.0068	0.0243	0.2167	0.0621	0.0040
S14	0.0033	0.0074	0.0032	0.0012	0.1801	0.0437	0.0013
S15	0.0159	0.0155	0.0137	0.0907	0.1988	0.0884	0.0018

Table S11 Chronic hazard quotient of metals to aquatic organisms at different sampling points

Sample	$HQ_{Chronic}$						
	Cd	Pb	Cu	Zn	Cr(Ⅵ)	Ni	Mn
S1	0.0732	0.3970	0.0456	0.2964	0.2902	0.4715	0.0216
S2	0.0350	0.2762	0.0257	0.0482	0.2245	0.4427	0.0242
S3	0.0614	0.8915	0.0274	0.0336	0.3306	0.3869	0.0209
S4	0.0158	0.5581	0.0276	0.1241	0.6287	0.3828	0.0352
S5	0.0756	0.6291	0.0314	0.0575	4.1251	0.5291	0.2267
S6	0.0761	0.4719	0.0165	0.2514	0.2266	0.3177	0.0197
S7	0.0132	0.0782	0.0191	0.0562	2.6181	0.4401	0.1331
S8	0.2297	39.3702	0.0325	0.1445	2.9985	0.6342	0.0993
S9	0.0145	0.3394	0.0206	0.0175	0.2916	0.2934	0.0222
S10	0.0015	0.0325	0.0203	0.0228	0.2138	0.6532	0.2865
S11	0.0249	0.5435	0.0306	0.0710	0.2972	0.2772	0.0433
S12	0.0110	0.5448	0.0457	0.1027	2.1029	0.3373	0.2219
S13	0.0248	0.1973	0.0225	0.0793	0.3164	0.3312	0.0486
S14	0.0149	0.1899	0.0107	0.0040	0.2629	0.2327	0.0156
S15	0.0077	0.2865	0.0222	0.1004	0.3120	0.3080	0.0175

Table S12 Pollution index of heavy metals in Fen River

Sample	Single factor pollution index							Integrated pollution index (*I*)
	Cd	Pb	Cu	Zn	Cr	Ni	Mn	
S1	0.1596	0.2558	0.1369	0.1155	0.1217	0.4440	0.0363	0.1814
S2	0.0762	0.1780	0.0772	0.0188	0.0941	0.4170	0.0407	0.1289
S3	0.1340	0.5745	0.0823	0.0131	0.1386	0.3644	0.0351	0.1917
S4	0.0344	0.3596	0.0829	0.0484	0.2635	0.3605	0.0592	0.1726
S5	0.1647	0.4054	0.0942	0.0224	1.7290	0.4983	0.3813	0.4708
S6	0.1658	0.3041	0.0495	0.0979	0.0950	0.2992	0.0331	0.1492
S7	0.0287	0.0504	0.0573	0.0219	1.0974	0.4145	0.2239	0.2706
S8	0.5007	25.3694	0.0976	0.0563	1.2568	0.5973	0.1670	4.0064
S9	0.0317	0.2187	0.0619	0.0068	0.1222	0.2763	0.0374	0.1079
S10	0.0032	0.0209	0.0609	0.0089	0.0896	0.6152	0.4820	0.1830
S11	0.0544	0.3502	0.0919	0.0277	0.1246	0.2611	0.0728	0.1404
S12	0.0239	0.3511	0.1371	0.0400	0.8814	0.3176	0.3731	0.3035
S13	0.0541	0.1271	0.0676	0.0309	0.1326	0.3119	0.0817	0.1151
S14	0.0326	0.1224	0.0321	0.0015	0.1102	0.2192	0.0262	0.0778
S15	0.0167	0.1846	0.0665	0.0391	0.1308	0.2901	0.0295	0.1082

References:

1. Schubauer-Berigan MK, Dierkes JR, Monson PD, et al. pH-dependent toxicity of cadmium, copper, nickel, lead and zinc to *Ceriodaphnia dubia*, *Pimephales promelas*, *Hyalella azteca* and *Lumbriculus variegatus*. *Environmental Toxicology and Chemistry*, **1993**, 12(7): 1261-1266.
2. Carlson SE, Rhodes PG, Ferguson MG. Docosahexaenoic acid status of preterm infants at birth and following feeding with human milk or formula. *American Journal of Clinical Nutrition*, **1986**, 44(6): 798-804.
3. Belanger SE, Farris JL, Cherry DS. Effects of diet, water hardness, and population source on acute and chronic copper toxicity to *Ceriodaphnia dubia*. *Archieves of Environmental Contamination and Toxicology*, **1989**, 18(4): 601-611.
4. Oris JT, Winner RW, Moore MV. A four-day survival and reproduction toxicity test for *Ceriodaphnia dubia*. *Environmental Toxicology and Chemistry*, **1991**, 10(2): 217-224.
5. Diamond JM, Koplish DE, Iii MM, et al. Evaluation of the water-effect ratio procedure for metals in a riverine system. *Environmental Toxicology & Chemistry*, **1997**, 16(3): 509-520.
6. Wang N, Mebane CA, Kunz JL, et al. Influence of dissolved organic carbon on toxicity of copper to a unionid mussel (*Villosa iris*) and a cladoceran (*Ceriodaphnia dubia*) in acute and chronic water exposures. *Environmental Toxicology and Chemistry*, **2011**,30(9): 2115-2125.
7. Nebeker AV, Cairns MA, Onjukka ST, et al. Effect of age on sensitivity of *Daphnia magna* to cadmium, copper and cyanazine. *Environmental Toxicology and Chemistry*, **1986**, 5(6):527-530.
8. Baird DJ, Barber I, Bradley M, et al. A comparative study of genotype sensitivity to acute toxic stress using clones of *Daphnia magna* straus. *Ecotoxicology and Environmental Safety*, **1991**, 21(3): 257-265.
9. Meador JP. The interaction of pH, dissolved organic carbon, and total copper in the determination of ionic copper and toxicity. *Aquatic Toxicology*, **1991**, 19(1): 13-31.

10. US EPA. Aquatic Life Ambient Freshwater Quality Criteria-Copper, EPA-822-R-07-001; United States Environmental Protection Agency Office of Water 4304T: Washington DC. 2007.
11. Zhou T, Cao Y, Qin L, et al. Application of biotic ligand model for the acute toxicity of copper to *Daphnia magna* in water of Liao River and Taihu Lake. *Environmental Science*, **2014**, 35(5):332-337.
12. Wang W, Chen S, Wu M, et al. Predicting copper to *Hypophthalmichthys molitrix* and *Ctenopharyngodon idellus* based on Biotic Ligand Model. *Environmental Sciences*, **2014**, 35(10): 3947-3951(in Chinese).
13. Chen S. Water quality criteria and ecological risk assessment of copper in Lancang River. M.S. Thesis, Kunming University of Science and Technology: Kunming. 2014.
14. Zhang YH, Zang WC, Qin LM, et al. Water quality criteria for copper based on the BLM approach in the freshwater in China. *PLOS One*, **2017**, 10.1371/journal.pone.0170105.
15. Khangarot BS, Ray PK. Correlation between heavy metal acute toxicity values in *Daphnia magna* and fish. *Bulletin of Environmental Contamination & Toxicology*, **1987**a, (38): 722-726.
16. Yim JH, Kim KW, Kim SD. Effect of hardness on acute toxicity of metal mixtures using *Daphnia magna*: prediction of acid mine drainage toxicity. *Journal of Hazardous Materials*, **2006,** 138 (1): 16-21.
17. Biesinger KE, Christensen GM. Effects of various metals on survival, growth, reproduction, and metabolism of *Daphnia magna*. *Journal of the Fisheries Research Board of Canada*, **1972**, 29(12): 1691-1700.
18. Chapman A G, Ota S, and Recht F. Effects of water hardness on the toxicity of metals to *Daphnia magna*. US EPA, Corvallis, OR:17 p. 1980.
19. Rathore RS, Khangarot BS. Effects of temperature on the sensitivity of sludge worm *Tubifex tubifex* müller to selected heavy metals. *Ecotoxicology Environmental Safety*, **2002**, 53(1): 27-36.
20. Qureshi SA, Saksena AB, & Singh VP. Acute toxicity of four heavy metals to benthic fish food organisms from the River Khan, Ujjain. *International Journal of Environmental Studies*, **1980**, 15(1): 59-61.
21. Spehar RL, Fiandt JT. Acute and chronic effects of water quality criteria-based metal mixtures on three aquatic species. *Environmental Toxicology and Chemistry*, **1986**, 5(10): 917-931.
22. Cooper NL, Bidwell JR, & Kumar A. Toxicity of copper, lead, and zinc mixtures to *Ceriodaphnia dubia* and *Daphnia carinata*. *Ecotoxicology & Environmental Safety*, **2009**, 72 (5): 1523-1528.
23. Hockett JR, Mount DR. Use of metal chelating agents to differentiate among sources of acute aquatic toxicity. *Environmental Toxicology and Chemistry,* **1996**, 15(10): 1687-1693.
24. Datta S, & Das RC. Influence of some abiotic environmental factors on acute toxicity of inorganic lead to *Cyprinus carpio var communis* (Linn.) and *Catla catla* (Ham.) in simulated toxic aquatic environment. *Toxicological & Environmental Chemistry*, **2003**, 85(4-6): 203-219.
25. Kapur K, Yadav NA. The effects of certain heavy metal salts on the development of eggs in common carp, *Cyprinus carpio* var. communis. *Clean-Soil, Air, Water*, **2010**, 10(5): 517-522.
26. Khangarot BS, Ray PK. Sensitivity of midge larvae of Chironomus tentans Fabricius (*Diptera chironomidae*) to heavy metals. *Bulletin of Environmental Contamination and Toxicology*, **1989**, 42 (3): 325-330.
27. Bailey HC, Liu DHW. *Lumbriculus variegatus*, a Benthic Oligochaete, as a Bioassay Organism. In: J.C. Eaton, P.R.Parrish, and A.C. Hendricks (Eds.), Aquatic Toxicology and Hazard Assessment, 3rd Synposium, ASTM STP 707, Philadelphia, 1980. PA: 205-215.
28. Wen RS, Zheng QM, Fang ZQ, et al. Acute toxicity of mercury and lead to grass carps and safety assessment. *Journal of Anhui Agricultural Sciences*, **2007**, 35 (16): 4863-4864, 4914. (in Chinese).
29. Pan TY, Fang SL, Yan YL, et al. Acute toxic effect of water-borne lead on six fishes from the upper reaches of the Yangtze River. *Freshwater Fisheries*, **2016**, (3): 34-39. (in Chinese).

30. Wang YQ, Zhang YQ, Zhao DQ. Effects of heavy metals cadmium, lead and zinc on the survival of *Carassius auratus* and *Misgurnus anguillicaudatus*. *Journal of Gansu Sciences*, **2003**, 15 (1): 35-38. (in Chinese).
31. Liao J. Distribution characterization of heavy metal pollution in Taihu Lake and the derivation of water quality criteria of lead in China. M.S. Thesis, Nanjing University: Nanjing, 2014. (in Chinese).
32. Chen WG, Guo ZJ, Deng PP, et al. Acute toxicity of three heavy metal ions to rose bitterling *Rhodeus ocellatus* Fry. *Fishries Science*, **2010**, 29 (02): 109-111. (in Chinese).
33. Anderson RL, Walbridge CT, Fiandt JT. Survival and growth of *Tanytarsus dissimilis* (Chironomidae) exposed to copper, cadmium, zinc, and lead. *Archives of Environmental Contamination & Toxicology*, **1980**, 9(3): 329.
34. Rachlin JW, Jensen TE, and Warkentine B. "growth response of the green alga (*Chlorella saccharophila*) to selected concentrations of the heavy metals Cd, Cu, Pb, and Zn." Trace substances in environmental health: proceedings of University of Missouri's... annual conference. 1982.
35. Liu Q. Study on water quality criteria for protecting aquatic organisms of lead and chromium in Taihu Lake. M.S. Thesis, Anhui Jianzhu University: Anhui. 2021.(in Chinese).
36. Rehwoldt R, Menapace LW, Nerrie B, et al. The effect of increased temperature upon the acute toxicity of some heavy metal ions. *Bulletin of Environmental Contamination and Toxicology*, **1972**, 8(2): 91-96.
37. Khangarot BS, Sehgal A, Bhasin MK. "Man and the Biosphere"-Studies on Sikkim Himalayas. Part 1: Acute toxicity of copper and zinc to common carp *Cyprinus carpio* (Linn.) in soft water. *Acta Hydrochimica et Hydrobiologica*, **1983,** 11(6): 667-673.
38. Tabata Y, Ishigure K, Higaki H, et al. Radiation induced copolymerization of fluorine containing monomers. *Journal of Macromolecular Science Chemistry*, **1969**, 4(4): 801-813.
39. Yan ZF, Li XF, Liu DQ, et al. Acute toxicity of zinc to native freshwater organisms under different water hardness. *Environmental Engineering*, **2019**, 257(11): 22-27. (in Chinese).
40. Li L, He YJ, Song KJ, et al. Derivation of water quality criteria of zinc to protect aquatic life in Taihu Lake and the associated risk assessment. *Journal of Environmental Management*, **2021**, 296:113175.
41. Christopher AM, Frank SD, Daniel PH. Acute toxicity of cadmium, lead, zinc, and their mixtures to stream-resident fish and invertebrates. *Environmental Toxicology and Chemistry*, **2012**, 31(6): 1334-1348.
42. Carlson AR, Nelson H, Hammermeister D. Development and validation of site-specific water quality criteria for copper. *Environmental Toxicology and Chemistry*, **1986**, 5(11): 997-1012.
43. Drost RJ, Singer AC. Constrained complexity generalized context-tree algorithms. IEEE/SP 14th Workshop on Statistical Signal Processing, 2007, 131-135.
44. Muyssen BT, Janssen CR. Multigeneration zinc acclimation and tolerance in *Daphnia magna*: implications for water-quality guidelines and ecological risk assessment. *Environmental Toxicology and Chemistry*, **2001**, 20(9): 2053-2060.
45. Muyssen BT, Janssen CR. Tolerance and acclimation to zinc of *Ceriodaphnia dubia*. *Environmental Pollution*, **2002**, 117(2): 301-306.
46. Wang X, Jin FF, Wang YA. Tropical ocean recharge nechanism for climate variability. Part II: A unified theory for decadal and ENSO modes. *Journal of Climate*, **2013**, 16(22): 3599-3616.
47. Ding TT, Du S, Zhang YH, et al. Hardness-dependent water quality criteria for cadmium and an ecological risk assessment of the Shaying river basin, China. *Ecotoxicology and Environmental Safety*, **2020**, 198:110666.

48. Bertramhart PE, Hart BA. Longevity and reproduction of *Daphnia pulex* exposed to cadmium contaminated food or water. *Environmental Pollution*, **1979**, 19(4): 295-305.
49. Elnabarawy MT, Welter AN, Robideau RR. Relative sensitivity of three Daphnid species to selected organic and inorganic chemicals. *Environmental Toxicology and Chemistry*, **2010**, 5(4): 393-398.
50. Hall WS, Paulson RL, Hall LW, et al. Acutetoxicity of cadmium and sodium pentachlorophenate to Daphnids and fish. *Bulletin of Environmental Contamination and Toxicology*, **1986**, 37(2): 308-316.
51. Suresh A, Sivaramakrishna B, Radhakrishnaiah K. Effect of lethal and sublethal concentrations of cadmium on energetics in the gills of fry and fingerlings of *Cyprinus carpio*. *Bulletin of Environmental Contamination & Toxicology*, **1993**, 51(6): 920-926.
52. Ghosal TK, Kaviraj A. Combined effects of cadmium and composted manure to aquatic organisms. *Chemosphere*, **2002**, 46(7): 1099-1105.
53. Shaw JR, Dempsey TD, Chen CY, et al. Comparative toxicity of cadmium, zinc, and mixtures of cadmium and zinc to Daphnids. *Environmental Toxicology and Chemistry*, **2006**, 25(1): 182-189.
54. Mohammed A, Agard JBR. Comparative sensitivity of three tropical Cladoceran species (*Diaphanosoma brachyurum*, *Ceriodaphnia rigaudii* and *Moinodaphnia macleayi*) to six chemicals. *Journal of Environmental Science and Health Part A*, **2006**, 41(12): 2713-2720.
55. Hatakeyama S, Yasuno HM. Effects of cadmium on the periodicity of parturition and brood size of *Moina macrocopa* (cladocera). *Environmental Pollution Series A, Ecological and Biological*, **1981**, 26(2): 111-120.
56. US EPA. Aquatic life ambient water quality criteria for Cadmium-2016. EPA-820-R-16-002. Office of Science and Technology, Health and Ecological Criteria Division. Washington, DC. 2016.
57. Taraldsen JE and Norberg-King TJ. New method for determining effluent toxicity using duckweed (*Lemna minor*). *Environmental Toxicology and Chemistry*, **1990**, 9: 761-767.
58. McCahon CP, Brown AF, Pascoe D. The effect of the acanthocephalan *Pomphorhynchus laevis* on the acute toxicity of cadmium to its intermediate host, the Amphipod *Gammarus pulex*. *Archives of Environmental Contamination and Toxicology*, **1988**, 17(2): 239-243.
59. He YJ. Study on water quality standard of heavy metal zinc and cadmium in Taihu Lake. M.S. Thesis, Anhui Jianzhu University: Anhui. 2021.(in Chinese).
60. Mount DI. Aquatic surrogates. In surrogates Peeies workshop report, Washington, DC: US EPA, 1982.
61. Call DJ, Brooke LT, Ahmad N, et al. Aquatic pollutant hazard assessments and development of a hazard prediction technology by quantitative structure-activity relationships. Second Quarterly Rep., U.S.EPA Cooperative Agreement No.CR 809234-01-0, Ct. For Lake Superior Environ. Stud, Univ. of Wisconsin, Superior, 1981, WI:74.
62. White B. Report of two toxicity evaluations conducted using hexavalent Chromium. Michigan DeP. Nat. Resour., Environ. Protection Bureau Point Sources Studies Section: 4.1979.
63. Mohammed A. Comparative sensitivities of the tropical Cladocera, *Ceriodaphnia rigaudii* and the temperate species *Daphnia magna* to seven toxicants. *Toxicology Environmental and Chemistry*, **2007**, 89(2): 347-352.
64. Shell Oi1 Co. Toxicity Tests with *Daphnia magna*: acute toxicity of five test materials to a newly-introduced strain of *D. magna* (Received October, 1984) with cover letter dated 103086. EPA/OTS Doc#86870000021 :16 p.(NTIS/OTS 0513367), 2000.
65. Enserink L, Luttmer W, & Maasdiepeveen H. Reproductive strategy of *Daphnia magna* affects the sensitivity of its progeny in acute toxicity tests. *Aquatic Toxicology*, **1990**, 17(l): 15-26.
66. Guilhermino L, Diamantino T, Silva MC. Acute toxicity test with *Daphnia magna*: an alternative to mammals

in the prescreening of chemical toxicity. *Ecotoxicology and Environmental Safety*, **2000**, 46(3): 357-362.

67. Mount DI, & Norberg UT. A seven-day life cycle Cladoceran toxicity test. *Environmental Toxicology & Chemistry*, **1984**, 3(3): 425-434.
68. Mount DI. Description of the toxicity tests performed on Cr^{6+} using Cladocerans. US EPA, Duluth, MN (Memo to C. Stephan, US EPA, Duluth, MN):7 p. 1982.
69. Jop KM, Rodgers JH, Price EE, et al. Renewal device for test solutions in *Daphnia* toxicity tests. *Bulletin of Environmental Contamination & Toxicology*, **1986**, 36(1): 95-100.
70. Bataccatalan Z, & Cairns J. Survival of *Daphnia pulex* under thermal-stress and sublethal concentration of chromate. *Kalikasan, the Philippine journal of biology*, **1977**, 6(1): 47-54.
71. Khangarot BS, Ray PK. Sensitivity of freshwater pulmonate snails, *Lymnaea luteola* L. to heavy metals. *Bulletin of Environmental Contamination and Toxicology*, **1988**, 41(2): 208-213.
72. Reynoldson TB, Rodriguez P, & Madrid MM. A comparison of reproduction, growth and acute toxicity in two populations of *Tubifex tubifex* (Müller, 1774) from the north American Great Lakes and northern Spain. *Hydrobiologia*, **1996**, 334(1-3): 199-206.
73. Khangarot BS. Toxicity of metals to a freshwater Tubificid worm, *Tubifex tubifex* (Muller). *Bulletin of Environmental Contamination & Toxicology*, **1991**, 46(6): 906-912.
74. Ewell WS, Gorsuch JW, Kringle RO, et al. Simultaneous evaluation of the acute effects of chemicals on seven aquatic species. *Environmental Toxicology and Chemistry*, **1986**, 5(9): 831-840.
75. Cowgill UM, Milazzo DP. The response of the three brood Ceriodaphnia test to fifteen formulations and pure compounds in common use. *Archives of Environmental Contamination and Toxicology*, **1991**, 21(1): 35-40.
76. Liang F. Risk assessment of heavy metals in typical watershed of China and derivation of hexavalent chromium water quality benchmark. Master's thesis of Nanjing: Nanjing University, Nanjing. 2011.
77. Zhang YL, Yuan J, Chen LP, et al. Joint toxicity experiment of three heavy metal on fry of *Carassius auratus*. *Hebei YuYe*, **2011**, (2): 24-27. (in Chinese).
78. Yang LH, Fang ZQ, Zheng WB. Safety assessment and acute toxicity of heavy metals to Crucian *Carassius auratus*. *Journal of south China normal university*, **2003**, (2): 101-105. (in Chinese).
79. Pickering QH, & Henderson C. The acute toxicity of some heavy metals to different species of warm water fishes. *Air & Water Pollution*, **1966**, 10(6): 453-463.
80. Adelman IR, Jr LLS, Siesennop GD. Acute toxicity of sodium chloride, pentachlorophenol, guthion, and hexavalent chromium to fathead minnows (*Pimephales promelas*) and goldfish (*Carassius auratus*). *Fisheries, Wildlife, and Conservation Biology*, **1976**, 33(2): 209-214.
81. Lv YP, Li XL, Jia XY. Study on the acute toxicity and joint toxicity of Cr^{6+}, Mn^{7+} and Hg^{2+} on the *Macrobrachium nipponense*. *Journal of Shanghai Fisheries University*, **2007**, (6): 549-554. (in Chinese).
82. Wong CK. Effects of chromium, copper, nickel, and zinc on survival and feeding of the Cladoceran *Moina macrocopa*. *Bulletin of Environmental Contamination and Toxicology*, **1992**, 49(4): 593-599.
83. Kazlauskiene N, Burba A and Svecevicius G. Acute toxicity of five galvanic heavy metals to Hydrobionts. *Ekonomika Ekologiia Cotsium*, **1994**, 1:33-36.
84. Meng FJ. Ecotoxicological effects of combined pollutions of perchlorate and chromium to *Daphnia carinata* and *Scenedesmus obliquus*. M.S. Thesis, South China Agricultural University: Guangdong, 2010. (in Chinese).
85. Chen HL, Pan G, Yan H, et al. Toxic effects of hexavalent chromium on the growth of Blue-Green microalgae. *Environmental Science*, **2003**, 2:13-18. (in Chinese).

86. St-Laurent D, Blaise C, Macquarrie P, et al. Comparative assessment of herbicide phytotoxicity to *Selenastrum capricornutum* using microplate and flask bioassay procedures. *Environmental Toxicology and Water Quality*, **1992**, 7(1): 35-48.
87. Liang F, Yang SG, Sun C. The acute toxicity of hexavalent chromium on *Pelteobagrus fulvidraco* Fry and Fingerling. *Journal of Agro-Environment Science*, **2010**, 29(9): 1665-1669. (in Chinese).
88. Keithly J, Brooker JA, DeForest DK, et al. Acute and chronic toxicity of nickel to a Cladoceran (*Ceriodaphnia dubia*) and an Amphipod (*Hyalella azteca*). *Environmental Toxicology and Chemistry*, **2004**, 23(3): 691-696.
89. Griffitt RJ, Luo J, Gao J, et al. Effects of particle composition and species on toxicity of metallic nanomaterials in aquatic organisms. *Environmental Toxicology and Chemistry*, **2008**, 27(9): 1972-1978.
90. Tsui MTK, Wang WX, and Chu LM. Influence of Glyphosate and its formulation (Roundup) on the toxicity and bioavailability of metals to *Ceriodaphnia dubia*. *Environmental Pollution*, **2005**, 138(1): 59-68.
91. Kuhn R, and Pattard M. Results of the harmful effects of water pollutants to green algae (*Scenedesmus subspicatus*) in the cell multiplication inhibition test. *Water Research*, **1990**, 24(1): 31-38.
92. Wang W. Toxicity tests of aquatic pollutants by using common Duck weed. *Environmental Pollution (Series B)*, **1986**, 11(1): 1-14.
93. Wang W. Toxicity of nickel to common Duckweed (*Lemna minor*). *Environmental Toxicology and Chemistry*, **1987**, 6(12): 961-967.
94. Khellaf N, and Zerdaoui M. Growth response of the Duckweed *Lemna minor* to heavy metal pollution. *Iranian Journal of Environmental Health Science & Engineering*, **2009**, 6(3): 161-166.
95. Bringmann G and Kuhn R. Comparative water-toxicological investigations on bacteria, algae, and Daphnia. TR-TS-002, Literature Research Company, Annandale, VA:26 p.1959.
96. Isherwood DM. Photoinduced toxicity of metals and PAHs to *Hyalella azteca*: UV-mediated toxicity and the effects of their photoproducts. M.S. Thesis, University of Waterloo, Canada: 266 p. 2009.
97. Liber K, Doig LE and White-Sobey SL.Toxicity of uranium, molybdenum, nickel, and arsenic to *Hyalella azteca* and *Chironomus dilutus* in water-only and spiked-sediment toxicity tests. *Ecotoxicology and Environmental Safety*, **2011**,74(5): 1171-1179.
98. Jindal R and Verma A. Heavy metal toxicity to *Daphnia pulex*. *Indian Journal of Environmental Health*, **1990**, 32(3): 289-292.
99. Khangarot BS, Ray PK and Chandra H. *Daphnia magna* as a model to assess heavy metal toxicity: comparative assessment with mouse system. *Acta hydrochimica et hydrobiologica*, **1987b**, 15(4): 427-432.
100. Call DJ, Brooke LT, Ahmad N, et al. Toxicity and metabolism studies with EPA (Environmental Protection Agency) priority pollutants and related chemicals in freshwater organisms. EPA 600/3-83-095, US EPA, Duluth, MN:120 p.1983.
101. Alsop D and Wood CM. Metal uptake and acute toxicity in zebrafish: common mechanisms across multiple metals. *Aquatic Toxicology*, **2011**, 105(3/4): 385-393.
102. Nebeker AV, Savonen C, and Stevens DG. Sensitivity of rainbow trout early life stages to nickel chloride. *Environmental Toxicology and Chemistry*, **1985**, 4(2): 233-239.
103. Buhl KJ, and Hamilton SJ. Relative sensitivity of early life stages of arctic grayling, Coho Salmon, and Rainbow Trout to nine inorganics. *Ecotoxicology and Environmental Safety*, **1991**, 22: 184-197.
104. Palawski D, Hunn JB, and Dwyer FJ. Sensitivity of young striped bass to organic and inorganic contaminants in fresh and saline waters. *Transactions of the American Fisheries Society*, **1985**, 114(5): 748-753.

105. Bentley RE, Heitmuller T, Sleight III BH, et al. Acute toxicity of nickel to Bluegill (*Lepomis macrochirus*), Rainbow Trout (*Salmo gairdneri*), and Pink Shrimp (*Penaeus duorarum*). US EPA, Criteria Branch, WA-6-99-1414-B, Washington, D.C. 1975.
106. Ding SR. Acute toxicities of vanadium, nickel and cobalt to several species of aquatic organisms. *Journal of Environmental Quality*, **1980**, 1: 17-21.
107. Rehwoldt R, Lasko L, Shaw C, et al. The acute toxicity of some heavy metal ions toward benthic organisms. *Bulletin of Environmental Contamination and Toxicology*, **1983**, 10(5): 291-294.
108. Virk S, and Sharma RC. Effect of nickel and chromium on various life stages of *Cyprinus carpio* Linn. *Indian Journal of Ecology*, **1995**, 22(2): 77-81.
109. Rao TS, Rao MS, and Prasad SBSK. Median tolerance limits of some chemicals to the fresh water fish "*Cyprinus carpio*". *Indian Journal of Environmental Health*, **1975**, 17(2): 140-146.
110. Blaylock BG, and Frank ML. A comparison of the toxicity of nickel to the developing eggs and larvae of Carp (*Cyprinus carpio*). *Bulletin of Environmental Contamination and Toxicology*, **1979**, 21(4-5): 604-611.
111. Rehwoldt R, Bida G, and Nerrie B. Acute toxicity of copper, nickel, and zinc ions to some Hudson River fish species. *Bulletin of Environmental Contamination and Toxicology*, **1971**, 6(5): 445-448.
112. Lu CH. Two heavy metal cobalt and nickel’s toxicity effects on two different loaches. M.S. Thesis, Henan Normal University: Henan, 2017. (in Chinese).
113. Shuhaimi-Othman M, Nadzifah Y, Nur-Amalina R, et al. Deriving freshwater quality criteria for copper, cadmium, aluminum and manganese for protection of aquatic life in Malaysia. *Chemosphere*, **2013**, 90(11): 2631-2636.
114. Eisentraeger A, Dott W, Klein J, et al. Comparative studies on algal toxicity testing using Fluorometric Microplate and Erlenmeyer Flask growth-inhibition assays. *Ecotoxicology and Environmental Safety*, **2003**, 54(3): 346-354.
115. Yan H, Pan G, Huo RL. Toxic effects of Cu, Zn and Mn on the inhibition of Chlorella pyrenoidosa's growth. *Acta Scientiae Circumstantiae*, **2001**, 21(3): 328-322. (in Chinese).
116. CCME (Canadian Council of Ministers of the Environment), Scientific criteria document for the development of the Canadian water quality guidelines for the protection of aquatic life—Manganese, PN 1601(ISBN 978-1-77202-058-8), 2019.
117. Cheng WD. Effects of exposure of manganese on ecological toxicology of *Ctenopharyngodon idellus* and *Cyprinus Carpio*. M.S. Thesis, University of Southwest, Chongqing, 2017. (in Chinese).
118. Wu S. Study on zebrafish indicating acid wastewater pollution in coal mine. M.S. Thesis, University of Guizhou: Guizhou, 2009. (in Chinese).
